# Longitudinal Exposure Patterns of Parent–Child Interaction Frequency and Emotional and Behavioral Difficulties and Neurodevelopmental Delays in Preschool Children: A Three-Wave Observational Study

**DOI:** 10.3390/bs16091504

**Published:** 2026-08-27

**Authors:** Dan Lin, Yanxia Wang, Zhiqing Chen, Qinfang Qian, Ping Ou, Jingmin Guo

**Affiliations:** Fujian Maternity and Child Health Hospital, College of Clinical Medicine for Obstetrics & Gynecology and Pediatrics, Fujian Medical University, Fuzhou 350001, China

**Keywords:** parent–child interaction frequency, preschool children, longitudinal exposure, emotional and behavioral difficulties, neurodevelopmental delay

## Abstract

Parent–child interaction frequency may vary during the transition into kindergarten and across activity content, but whether persistent or repeated low-frequency interaction is associated with preschool children’s emotional-behavioral and neurodevelopmental outcomes remains unclear. We examined whether three-wave overall and content-domain trajectories, together with cumulative low-level exposure at the overall, content-domain, and activity levels, were associated with final-follow-up outcomes. Among 526 children enrolled at baseline, 419 completed all three waves. Low exposure was defined as a score below the wave-specific 25th percentile, and adjusted logistic regression models estimated associations. Compared with the persistently normal trajectory, the persistently low overall trajectory was associated with higher odds of abnormal total emotional and behavioral difficulties (aOR = 3.46, 95% CI 1.01–11.86); each additional low-exposure wave was also associated with higher odds (aOR = 1.54, 95% CI 1.02–2.33), with similar findings for conduct problems. Nominal content-domain associations were concentrated in story-reading and environmental interaction trajectories, while nominal activity-level associations involved reading/picture-book viewing, learning about nature/animals/plants, and physical activity. Neurodevelopmental associations were fewer and less stable. By jointly characterizing temporal pattern, repeated low exposure, and activity content, this study illustrates the value of repeated and content-specific assessment alongside a single overall interaction measure. These dimensions may inform assessment in parenting support and early-childhood services, but intervention evidence is required before causal or activity-specific recommendations can be made.

## 1. Introduction

### 1.1. Parent–Child Interaction as a Developmental Context

The preschool period is an important stage for the rapid development of children’s emotional regulation, behavioral control, social adaptation, language communication, and neurodevelopment (Black et al., 2017). During this period, children gradually move from life contexts dominated by family care into kindergarten and peer-group environments, and family experiences and group experiences begin to jointly influence early child development. Previous research on early childhood development and the Nurturing Care Framework has emphasized that responsive caregiving and early learning opportunities are important environmental foundations for supporting healthy child development (Black et al., 2017; Britto et al., 2017; World Health Organization et al., 2018). As an observable process in the family environment that is closely connected with children’s daily life experiences, parent–child interaction provides children with repeated development-promoting stimulation, including language input, emotional responses, behavioral guidance, rule learning, joint attention, and exploratory feedback (Sameroff, 2009).

Parent–child interaction does not merely refer to whether parents accompany children, nor is it simply a family-background indicator. Different forms of parent–child interactive activities may carry different developmental functions. Parent–child shared reading and storytelling usually involve vocabulary input, narrative understanding, emotional discussion, and joint attention. Cognitive games, number-concept learning, and literacy activities in daily life involve task persistence, rule understanding, and adult feedback. Physical activity, handicrafts, and self-care activities provide opportunities for children to practice motor control, turn-taking, and stepwise task completion. Discussions of surrounding events and learning about nature, animals, and plants embed language exchange, observation, explanation, and shared exploration in daily life. Parent–child interaction frequency may therefore reflect both the amount of family interaction and the developmental opportunities children receive repeatedly through different types of interaction (Bornstein et al., 2020; Flack et al., 2018; Ip et al., 2018; Mol et al., 2008; Purpura et al., 2020; Wu et al., 2020; Xie et al., 2018).

### 1.2. Need for a Longitudinal and Content-Specific Approach

Previous studies have shown that parent–child interaction, responsive caregiving, the home learning environment, parent–child shared reading, parent–child play, and early parenting interventions are related to children’s language ability, social-emotional development, behavioral adaptation, executive function, and early learning (Jeong et al., 2021; Landry et al., 2008; Mingebach et al., 2018; Schneider et al., 2022; van IJzendoorn et al., 2023; Yousafzai et al., 2014). Related studies published in recent years have linked parenting processes with preschool adjustment and social-emotional competence and home literacy activities with early literacy development (Bergman Deitcher et al., 2024; Boise & Knoche, 2024; Wang et al., 2023). However, many studies have used cross-sectional designs or assessed the family environment and parent–child interaction at only a single time point, making it difficult to distinguish temporary low interaction frequency, persistent low interaction frequency, and patterns that change over time (Wu et al., 2020). For children during the transition after kindergarten entry, parent–child interaction frequency may not be static but may change with family routines, children’s adjustment, and caregiving arrangements.

Parent–child interaction frequency may change across consecutive assessments and should therefore be examined longitudinally. Repeated low-frequency interaction may also have cumulative importance. Children who remain at a lower interaction level across several waves may differ from those who do so at only one wave. Considering both change patterns and cumulative low-level exposure can therefore provide information about children’s family experiences over time that a single assessment cannot capture (Gadeyne et al., 2004; Hollenstein et al., 2004; Ogg & Anthony, 2019; Smeekens et al., 2007).

In addition, parent–child interaction frequency itself may not fully reflect differences in interactive content. The same interaction frequency may correspond to different activity types, communication forms, and developmental stimulation. Some interactions place greater emphasis on language input and narrative communication (Flack et al., 2018; Hamilton et al., 2016; Mol et al., 2008; Puglisi et al., 2017; Xie et al., 2018). Other interactions place greater emphasis on rule learning, cognitive participation, physical activity, or environmental exploration (Bornstein et al., 2020; Ebert et al., 2020; Ip et al., 2018; Li et al., 2022; Linberg et al., 2020; Purpura et al., 2020; Wu et al., 2020). If parent–child interaction is treated only as a homogeneous family exposure, differences in the associations between different interactive content and children’s emotional and behavioral difficulties or neurodevelopmental delays may be obscured. Longitudinal analyses should therefore account for variation in parent–child interaction content.

### 1.3. Research Questions and Hypotheses

Accordingly, this study addressed two research questions. First, were three-wave overall parent–child interaction trajectories and cumulative low-level exposure associated with emotional and behavioral difficulties and screening-positive neurodevelopmental delays at final follow-up? Second, were content-domain trajectories and cumulative low-level exposure, as well as activity-level cumulative low-level exposure, associated with these outcomes? We hypothesized that a persistently low overall trajectory and greater cumulative low-level exposure would be associated with higher odds of emotional and behavioral difficulties and screening-positive neurodevelopmental delays. Because prior evidence was insufficient to specify directional hypotheses for individual content domains and activities, those analyses were exploratory.

## 2. Materials and Methods

### 2.1. Study Design and Participants

This was a three-wave longitudinal observational study, reported in accordance with the Strengthening the Reporting of Observational Studies in Epidemiology (STROBE) statement (Vandenbroucke et al., 2007; von Elm et al., 2007). Participants were preschool children enrolled in participating kindergartens in September 2025 and their guardians. Eligible children were at least 36 months old and younger than 48 months at the baseline assessment. The main inclusion criteria were voluntary participation of the child and guardian; the family planned to reside locally long term, and the child had no plan to change kindergarten during preschool education; the child had no congenital disease, birth defect, disability, or severe systemic disease; and the guardian provided informed consent and cooperated with follow-up assessments. Because participation was voluntary, no information was collected from families that did not take part. The study protocol was approved by the Ethics Committee of Fujian Maternity and Child Health Hospital (approval number: 2023KY129), and all children were enrolled after guardian informed consent. The participant follow-up flow is presented in Supplementary Figure S1.

### 2.2. Data Collection and Quality Control

The survey was organized by the project team and field investigators, and questionnaires were mainly completed by the child’s parents or primary caregivers. Data collection included electronic questionnaires completed after scanning a QR code and paper or electronic questionnaires completed face to face on site. The three assessment waves were the baseline assessment at kindergarten entry in September 2025, the first follow-up at the end of the first semester in December 2025, and the final follow-up in April 2026. Because neurodevelopmental assessment requires matching the questionnaire age version to the child’s actual age in months, the database recorded each child’s date of birth, survey date, age in months, questionnaire age version, and domain results. Quality control procedures included identity verification, completeness checks for key variables, logical range checks, verification that the questionnaire age version matched the child’s actual age in months, and confirmation through reserved contact information when necessary.

### 2.3. Measures

#### 2.3.1. Parent–Child Interaction Frequency

Parent–child interaction frequency was assessed using the Chinese Parent–Child Interaction Scale (CPCIS), a parent-reported instrument used to assess how often parents and preschool children in Chinese families jointly participate in interactive activities. Development and validation work for CPCIS reported a person separation reliability of 0.81 and a Cronbach’s alpha of 0.82 (Ip et al., 2018). The present study used the 12-item version applied in research on Chinese preschool children; that Shanghai kindergarten study reported an internal consistency reliability of 0.88 for CPCIS (Wu et al., 2020). The 12 interactive activities were parent–child reading/picture-book viewing, teaching number concepts, drawing/painting, cognitive games, learning characters in daily life, listening to or singing songs, poems, or nursery rhymes, storytelling, handicrafts, physical activity, self-care skills, discussing surrounding events, and learning about nature, animals, or plants. The four content dimensions were knowledge learning (items 2, 4, and 5), story reading (items 1, 6, and 7), physical/cultural activities (items 3, 8, 9, and 10), and environmental interaction (items 11 and 12).

#### 2.3.2. Emotional and Behavioral Difficulties

Children’s emotional and behavioral difficulties were assessed using the parent version of the Strengths and Difficulties Questionnaire (SDQ). The SDQ includes 25 items and yields five subscales: emotional symptoms, conduct problems, hyperactivity/inattention, peer problems, and prosocial behavior (Goodman, 1997, 2001). The first four problem-domain subscales are summed to generate a total emotional and behavioral difficulties score, while prosocial behavior is scored separately. Du et al. (2008) established norms and psychometric data for the parent, teacher, and self-report versions of the SDQ in a Chinese sample of children and adolescents aged 3–17 years; Chinese validation studies have reported internal consistency coefficients as high as 0.83 and provided evidence for structural validity, convergent validity, discriminant validity, and normative data. Additional validation work has supported the use of the SDQ in preschool-aged children (Croft et al., 2015). Binary outcomes were defined according to Chinese parent-version abnormal banding cutoffs: total difficulties score > 16, emotional symptoms > 4, conduct problems > 3, hyperactivity/inattention > 7, peer problems > 5, and prosocial behavior < 5.

#### 2.3.3. Neurodevelopmental Screening-Positive Delay

Neurodevelopmental screening-positive delay was assessed using the Chinese version of the Ages and Stages Questionnaires, Third Edition (ASQ-3). The ASQ-3 is a parent-completed child developmental assessment tool used to evaluate age-related milestones in five domains: communication, gross motor, fine motor, problem-solving, and personal-social abilities (Squires et al., 2009). The Chinese national norming study established norms and psychometric data for children aged 1–66 months; related research reported an overall Cronbach’s alpha of 0.80, an inter-rater correlation coefficient of 0.80, a test–retest correlation coefficient of 0.80, and sensitivity and specificity data (Wei et al., 2015). Additional validation work has supported the use of ASQ-3 developmental screening (Schonhaut et al., 2013). The appropriate ASQ-3 questionnaire age version was matched to each child’s actual age in months at each survey. Standardized scoring procedures and age-version thresholds were used to define communication delay, gross motor delay, fine motor delay, problem-solving delay, personal-social delay, and screening-positive delay in any neurodevelopmental domain.

### 2.4. Construction of Longitudinal Parent–Child Interaction Frequency Exposures

At each assessment wave, the overall parent–child interaction frequency score, the four content-domain scores, and the 12 activity-level scores were categorized as low-level exposure if they were below the wave-specific 25th percentile of the sample distribution. Because the cutoff was defined within each wave, low-level exposure indicates a relative position within the study sample rather than an absolute lack of interaction.

**Figure 1:**
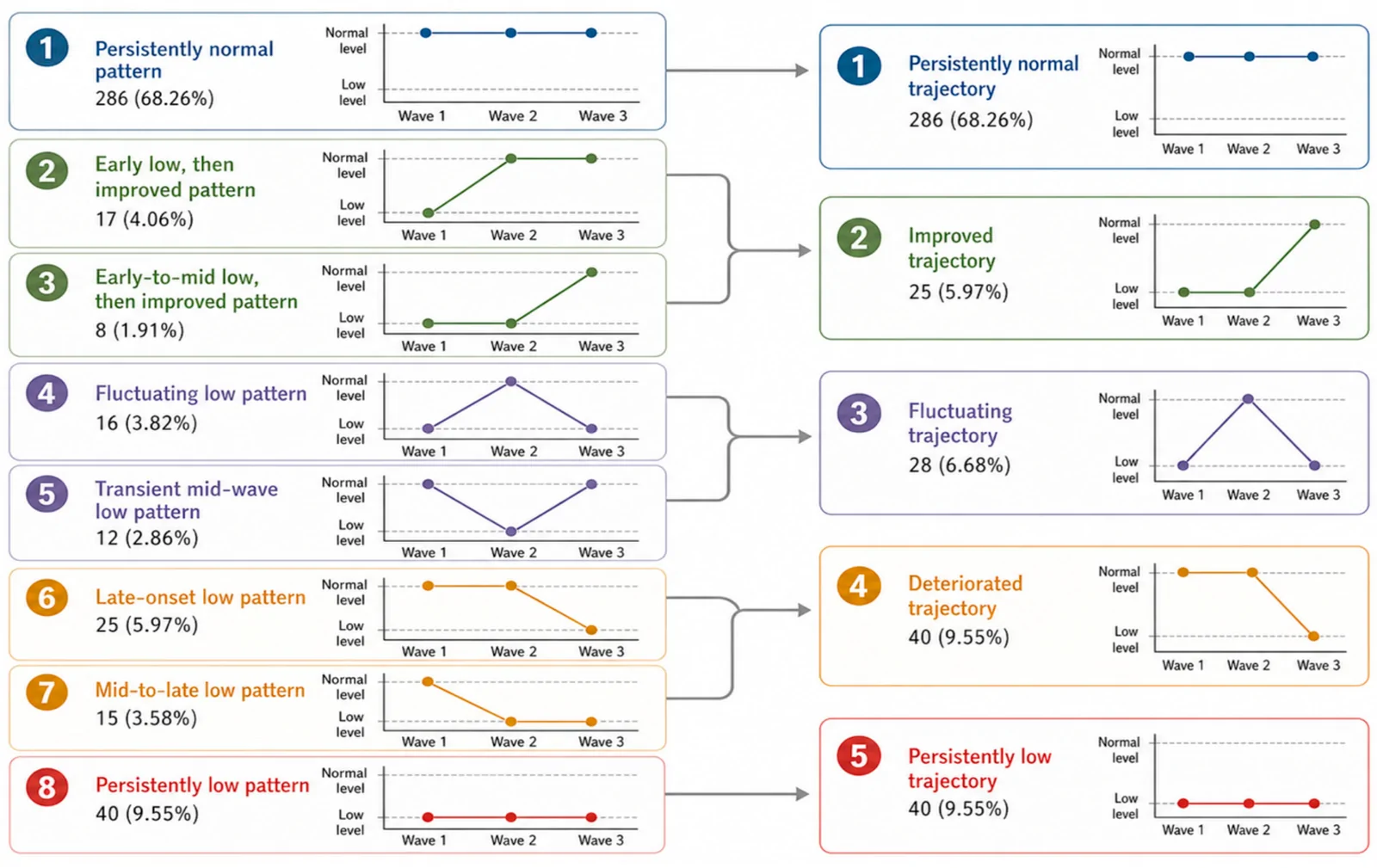
Construction of three-wave parent–child interaction frequency patterns and longitudinal trajectory groups. Note: Low indicates below the wave-specific 25th percentile of parent–child interaction frequency; normal indicates not below the wave-specific 25th percentile.

For the overall score and the four content-domain scores, the binary low-level exposure status across the three waves first generated eight original patterns. These were then merged into five longitudinal trajectory groups according to the direction of change: Normal-Normal-Normal was classified as persistently normal; Low-Low-Low as persistently low; Low-Normal-Normal and Low-Low-Normal as improved; Normal-Normal-Low and Normal-Low-Low as deteriorated; and Low-Normal-Low and Normal-Low-Normal as fluctuating. Cumulative low-level exposure was coded as 0, 1, 2, or 3, indicating the number of waves at which the child was classified as having low-level parent–child interaction frequency. The activity-level analyses used the same cumulative low-level exposure count for each of the 12 specific interactive activities. The trajectory-group construction is shown in Figure 1.

### 2.5. Covariates

Candidate baseline covariates included child sex, residence, preschool ownership, only-child status, maternal education, annual household income, preterm birth, low birth weight, history of neonatal intensive care unit admission, breastfeeding history, maternal pregnancy risk, and any perinatal risk. These covariates were collected at the baseline assessment and were not remeasured at the two follow-up assessments. Covariate selection was based on the study design and the potential confounding structure rather than univariable screening (VanderWeele & Shpitser, 2011). The primary adjusted model included child sex, residence, preschool ownership, only child status, maternal education, annual household income, and any perinatal risk; the fully adjusted sensitivity model additionally included preterm birth, low birth weight, history of neonatal intensive care unit admission, breastfeeding history, and maternal pregnancy risk.

### 2.6. Statistical Analysis

Continuous variables were evaluated graphically and with normality tests. Non-normally distributed variables are presented as medians and interquartile ranges, and categorical variables as *n* (%). Across-wave comparisons used the Friedman test for continuous variables and Cochran’s Q test for binary variables. Final-follow-up outcome proportions were compared across baseline characteristic categories using Fisher’s exact test.

To examine whether overall parent–child interaction frequency was associated with final-follow-up emotional-behavioral and neurodevelopmental outcomes, logistic regression models represented overall interaction using the five-category trajectory (persistently normal as the reference) and the cumulative number of low-exposure waves (per additional wave). To examine whether these associations differed according to interaction content, the same exposure representations were analyzed for the four content domains, while activity-level analyses used cumulative low-level exposure for each of the 12 activities. Trajectory contrasts characterized temporal patterns, whereas cumulative counts assessed repeated low-level exposure. Models estimated adjusted odds ratios (aORs) and 95% confidence intervals using the primary covariate set to limit overfitting in sparse outcome strata. Fully adjusted and Firth penalized models evaluated the robustness of overall and content-domain findings.

When zero-event cells or model non-convergence precluded stable estimation, estimates were not reported (Heinze & Schemper, 2002; Peduzzi et al., 1996; Vittinghoff & McCulloch, 2007). *p* values were not adjusted for multiple comparisons; content-domain, activity-level, and post hoc single-wave findings were therefore considered nominal and were interpreted with their effect estimates and 95% confidence intervals. Post hoc analyses modeled low-level exposure separately at Wave 1, Wave 2, and Wave 3. Overall-interaction models covered all final-follow-up emotional-behavioral and neurodevelopmental outcomes, whereas content-domain and activity-level models were limited to exposure-outcome pairs with *p* < 0.05 in the corresponding longitudinal analysis. All post hoc models used the primary covariates. These post hoc analyses examined whether longitudinal associations were detectable at individual waves. Tests were two-sided, and *p* values were reported to three decimal places, with *p* < 0.001 reported when applicable. Statistical analyses and figure preparation were performed using R 4.5.2, with the dplyr (version 1.2.1), ggplot2 (version 4.0.3), officer (version 0.7.4), and flextable (version 0.9.11) packages (Gohel et al., 2026; Gohel & Skintzos, 2026; R Core Team, 2025; Wickham, 2016; Wickham et al., 2026).

## 3. Results

### 3.1. Participants and Repeated Assessments

The analytic database included 526 newly enrolled preschool children at baseline. A total of 419 children had complete repeated measurements across the three waves and were included in the repeated-assessment trajectory analyses. Median child age increased from 41.77 months at kindergarten entry to 44.77 months at the end-of-first-semester follow-up and 48.77 months at the final follow-up (*p* < 0.001). Table 1 summarizes the repeated assessments.

The overall parent–child interaction frequency score was stable across waves, whereas story-reading interaction frequency, physical/cultural interaction frequency, environmental interaction frequency, and low-level overall parent–child interaction frequency differed across waves. At the final follow-up, 38 children (9.07%) had abnormal total emotional and behavioral difficulties, and 36 children (8.59%) had screening-positive delay in at least one neurodevelopmental domain.

Neither abnormal total emotional and behavioral difficulties nor screening-positive delay in any neurodevelopmental domain differed significantly across baseline characteristic categories. Table 2 presents participant characteristics and final outcomes.

### 3.2. Overall Parent–Child Interaction Frequency Trajectories and Cumulative Low-Level Exposure in Relation to Emotional and Behavioral Difficulties and Neurodevelopmental Delays

**Figure 2:**
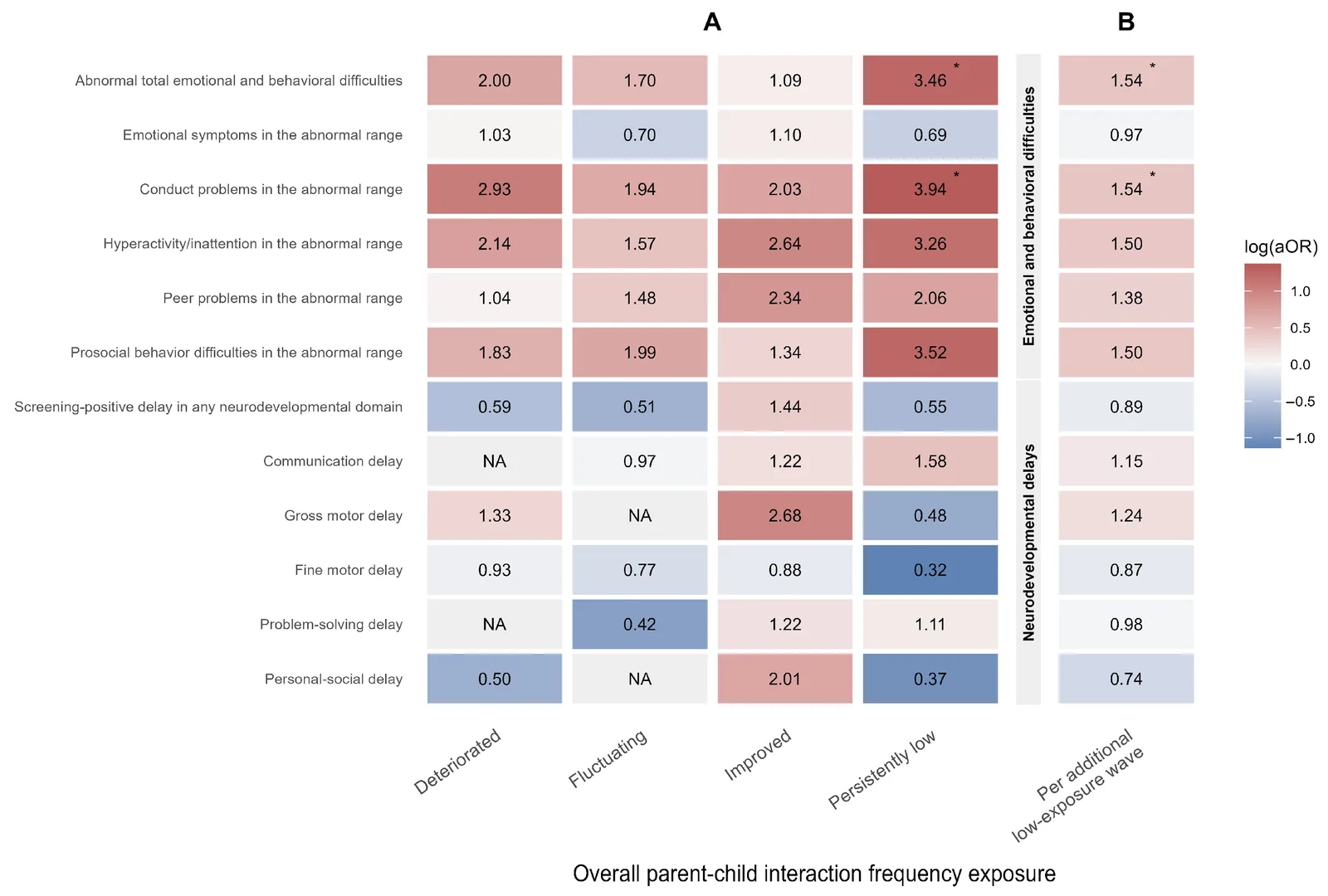
Adjusted associations of overall parent–child interaction frequency trajectories and cumulative low-level exposure with emotional and behavioral difficulties and neurodevelopmental delays. Note. Panel (**A**) shows trajectory contrasts using the persistently normal trajectory as the reference group; Panel (**B**) shows cumulative low-level exposure modeled per additional low-exposure wave. Cells show aORs from primary adjusted logistic regression models. Colors represent log(aORs). Asterisks indicate raw *p* < 0.05. NA indicates that the estimate was not reported because of an unstable model estimate. Complete numeric estimates are shown in Supplementary Table S1.

Compared with children in the persistently normal trajectory, children in the persistently low trajectory had higher odds of abnormal total emotional and behavioral difficulties (aOR = 3.46, 95% CI 1.01–11.86, *p* = 0.049). Each additional wave of low-level overall parent–child interaction frequency exposure was also associated with higher odds of abnormal total emotional and behavioral difficulties (aOR = 1.54, 95% CI 1.02–2.33, *p* = 0.041). Neither overall trajectory pattern nor cumulative low-level exposure was associated with screening-positive delay in any neurodevelopmental domain. Figure 2 and Supplementary Table S1 present the overall interaction results.

For specific emotional and behavioral difficulties and neurodevelopmental delays, the overall parent–child interaction frequency trajectory was associated with conduct problems in the abnormal range: children in the persistently low trajectory had higher odds than children in the persistently normal trajectory (aOR = 3.94, 95% CI 1.13–13.69, *p* = 0.031). For cumulative low-level exposure, each additional wave of low-level overall parent–child interaction frequency exposure was associated with higher odds of conduct problems in the abnormal range (aOR = 1.54, 95% CI 1.02–2.32, *p* = 0.039). No statistically significant association was observed for the specific neurodevelopmental delays.

### 3.3. Parent–Child Interaction Content-Domain Trajectories and Cumulative Low-Level Exposure in Relation to Emotional and Behavioral Difficulties and Neurodevelopmental Delays

**Figure 3:**
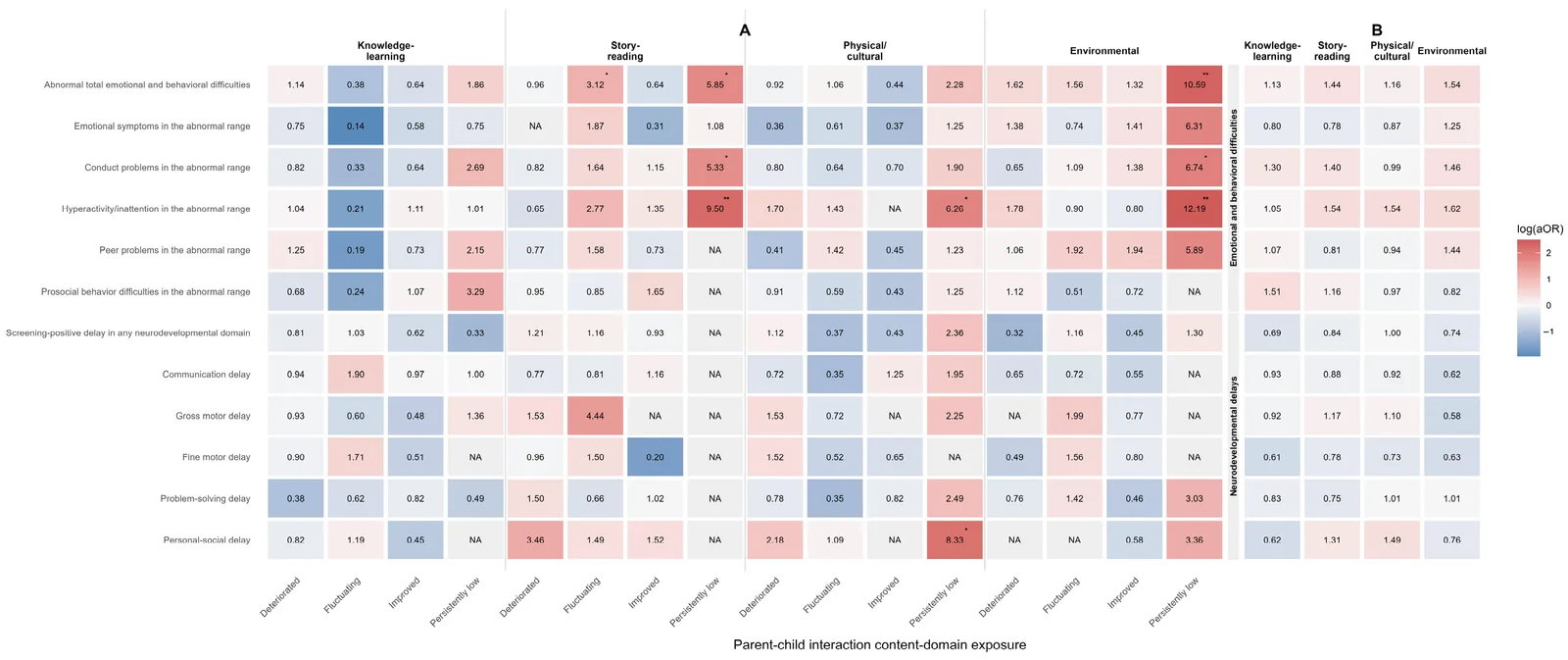
Adjusted associations of parent–child interaction content-domain trajectories and cumulative low-level exposure with emotional and behavioral difficulties and neurodevelopmental delays. Note. Panel (**A**) shows trajectory contrasts within each parent–child interaction content domain using the corresponding persistently normal trajectory as the reference group; Panel (**B**) shows cumulative low-level exposure modeled per additional low-exposure wave within each content domain. Cells show aORs from primary adjusted logistic regression models; colors represent log(aORs). Asterisks indicate raw *p* values: * *p* < 0.05 and ** *p* < 0.01. NA indicates that the estimate was not reported because of an unstable model estimate. Complete numeric estimates are shown in Supplementary Table S2.

Children in the fluctuating story-reading trajectory (aOR = 3.12, 95% CI 1.04–9.32, *p* = 0.042), persistently low story-reading trajectory (aOR = 5.85, 95% CI 1.20–28.60, *p* = 0.029), and persistently low environmental interaction trajectory (aOR = 10.59, 95% CI 1.89–59.39, *p* = 0.007) had higher odds of abnormal total emotional and behavioral difficulties than children in the corresponding persistently normal trajectory. No content-domain cumulative low-level exposure was associated with either abnormal total emotional and behavioral difficulties or screening-positive delay in any neurodevelopmental domain. Figure 3 and Supplementary Table S2 present the content-domain results.

For specific emotional and behavioral difficulties and neurodevelopmental delays, content-domain trajectory associations were observed primarily for persistently low story-reading, physical/cultural, and environmental interaction frequency trajectories. Children in the persistently low story-reading trajectory had higher odds of conduct problems (aOR = 5.33, 95% CI 1.08–26.25, *p* = 0.040) and hyperactivity/inattention (aOR = 9.50, 95% CI 1.83–49.40, *p* = 0.007). Children in the persistently low environmental interaction trajectory had higher odds of conduct problems (aOR = 6.74, 95% CI 1.22–37.23, *p* = 0.029) and hyperactivity/inattention (aOR = 12.19, 95% CI 2.00–74.39, *p* = 0.007). Persistently low physical/cultural interaction frequency was associated with hyperactivity/inattention (aOR = 6.26, 95% CI 1.41–27.86, *p* = 0.016) and personal-social delay (aOR = 8.33, 95% CI 1.07–64.93, *p* = 0.043). No content-domain cumulative measure was associated with a specific emotional-behavioral or neurodevelopmental outcome.

### 3.4. Specific Parent–Child Interactive Activities in Relation to Emotional and Behavioral Difficulties and Neurodevelopmental Delays

**Figure 4:**
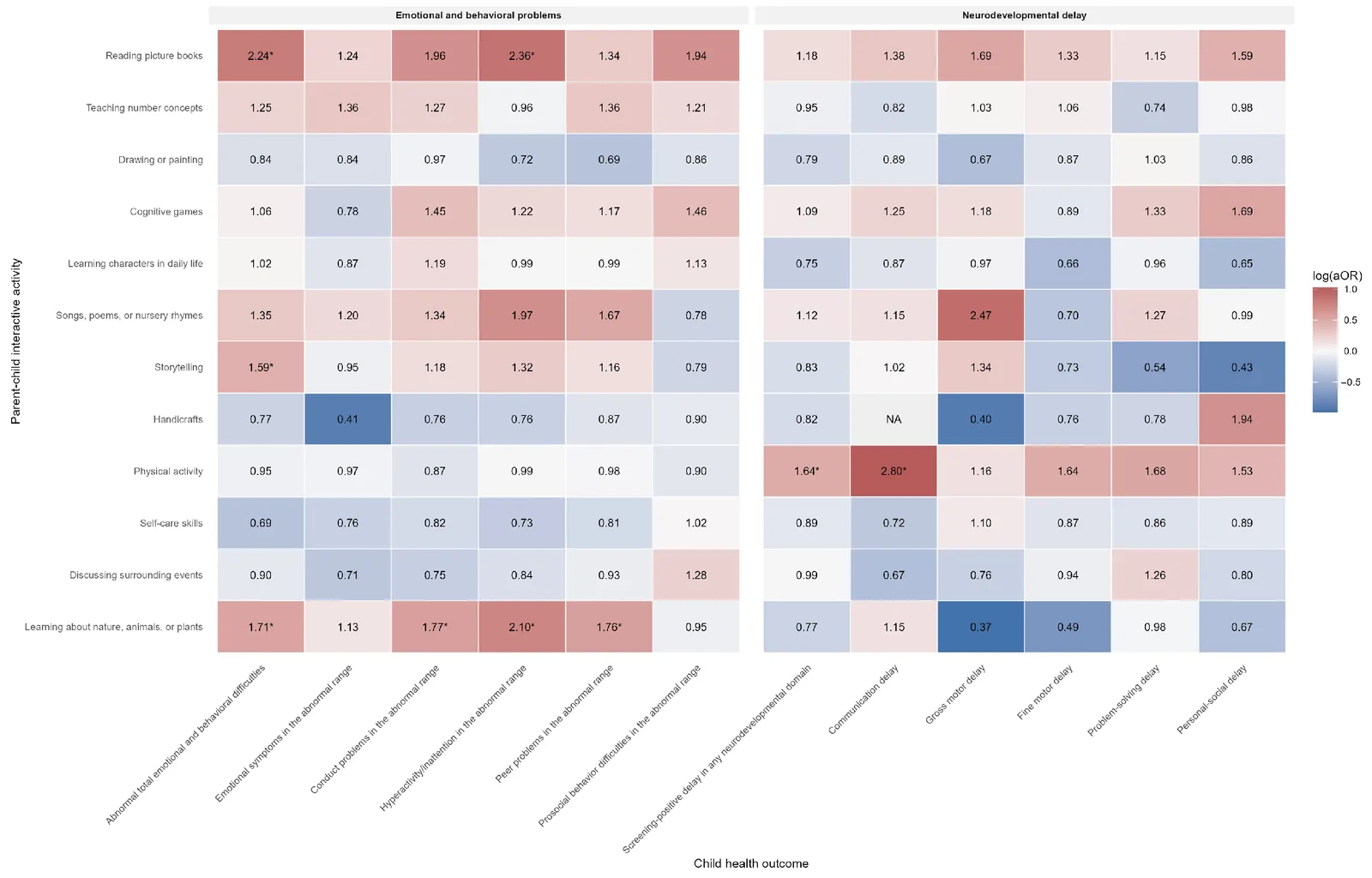
Adjusted associations of cumulative low-level exposure to specific parent–child interactive activities with emotional and behavioral difficulties and neurodevelopmental delays. Note. Cells show aORs from primary adjusted logistic regression models per additional wave of low-level exposure to the specific activity. Colors represent log(aORs). Asterisks indicate raw *p* < 0.05. NA indicates that the estimate was not reported because of an unstable model estimate. Complete numeric estimates are shown in Supplementary Table S3.

Each additional wave of low-level exposure to reading picture books (aOR = 2.24, 95% CI 1.05–4.75, *p* = 0.036) or to learning about nature, animals, or plants (aOR = 1.71, 95% CI 1.02–2.88, *p* = 0.043) was associated with higher odds of abnormal total emotional and behavioral difficulties. Children with additional waves of low-level physical activity exposure had higher odds of screening-positive delay in any neurodevelopmental domain (aOR = 1.64, 95% CI 1.03–2.62, *p* = 0.039). Figure 4 and Supplementary Table S3 present the activity-level results.

Children with additional waves of low-level exposure to learning about nature, animals, or plants had higher odds of conduct problems (aOR = 1.77, 95% CI 1.06–2.97, *p* = 0.030), hyperactivity/inattention (aOR = 2.10, 95% CI 1.20–3.67, *p* = 0.010), and peer problems (aOR = 1.76, 95% CI 1.02–3.04, *p* = 0.042). Children with additional waves of low-level exposure to reading picture books had higher odds of hyperactivity/inattention (aOR = 2.36, 95% CI 1.03–5.39, *p* = 0.041). The statistically significant neurodevelopmental screening associations both involved physical activity exposure: screening-positive delay in any neurodevelopmental domain (aOR = 1.64, 95% CI 1.03–2.62, *p* = 0.039) and communication delay (aOR = 2.80, 95% CI 1.35–5.82, *p* = 0.006).

### 3.5. Additional Analyses

#### 3.5.1. Sensitivity Analyses Using Alternative Model Specifications

In brief, the overall cumulative low-level exposure association with abnormal total emotional and behavioral difficulties remained statistically significant in the Firth penalized model but was attenuated in the fully adjusted model. The overall persistently low trajectory estimate for abnormal total emotional and behavioral difficulties was attenuated in both sensitivity models. The trajectory associations of fluctuating story-reading, persistently low story-reading, and persistently low environmental interaction frequency with abnormal total emotional and behavioral difficulties remained statistically significant in both sensitivity models. For specific emotional and behavioral difficulties and neurodevelopmental delays, sensitivity findings were concentrated in conduct problems and hyperactivity/inattention for persistently low story-reading, environmental interaction, or physical/cultural interaction frequency; screening-positive delay in any neurodevelopmental domain showed no statistically significant association in sensitivity analyses. Sensitivity analyses using fully adjusted logistic regression and Firth penalized logistic regression are provided in Supplementary Tables S4 and S5.

#### 3.5.2. Post Hoc Single-Wave Analyses

At Wave 2, low overall interaction was associated with abnormal total emotional and behavioral difficulties (aOR = 3.31, 95% CI 1.31–8.38, *p* = 0.011), conduct problems (aOR = 2.86, 95% CI 1.15–7.14, *p* = 0.024), and hyperactivity/inattention (aOR = 4.18, 95% CI 1.45–12.09, *p* = 0.008). No association reached *p* < 0.05 at Wave 1 or Wave 3, and no other Wave 2 estimate did. Post hoc analyses examined low overall parent–child interaction separately at each wave across all emotional-behavioral and neurodevelopmental outcomes (Supplementary Table S6).

Low story-reading at Wave 2 was associated with abnormal total difficulties and hyperactivity/inattention. At the same wave, low-frequency reading of picture books showed the same pattern, while low-frequency learning about nature, animals, or plants was associated with total difficulties, hyperactivity/inattention, and peer problems. Low physical/cultural interaction at Wave 3 was associated with hyperactivity/inattention. For neurodevelopmental outcomes, low physical activity was associated with screening-positive delay in any domain at Wave 3 and with communication delay at Wave 1. Several of the longitudinal associations were not observed in any single-wave model. Some estimates were imprecise because the low-exposure groups or the numbers of outcome events were small. Supplementary Table S7 presents the single-wave results for the selected content-domain and activity-level pairs.

## 4. Discussion

### 4.1. Principal Findings

Using three-wave follow-up data from newly enrolled preschool children, this study examined the associations of longitudinal exposure patterns of parent–child interaction frequency and different interaction contents with children’s emotional and behavioral difficulties and neurodevelopmental delays. The main finding was that lower longitudinal exposure to parent–child interaction frequency was mainly related to children’s emotional and behavioral difficulties, especially abnormal total emotional and behavioral difficulties, conduct problems, and hyperactivity/inattention. In contrast, findings related to neurodevelopmental delays were fewer and less stable. After parent–child interaction content was further decomposed, trajectory patterns of storytelling/reading-related interaction, environmental interaction, and some physical or cultural activities showed more concentrated associations with children’s emotional and behavioral difficulties. At the level of specific parent–child interactive activities, lower-frequency reading/picture-book viewing, learning about nature/animals/plants, and physical activity may correspond to different types of children’s emotional and behavioral difficulties or neurodevelopmental delay risks. These findings support understanding parent–child interaction frequency as a family exposure with temporal structure and content heterogeneity, rather than as a single-time-point or single-total-amount family background indicator.

### 4.2. Interpretation of Temporal and Outcome Patterns

These findings are consistent with proximal process theory and the transactional model of child development (Black et al., 2017; Britto et al., 2017; Sameroff, 2009; World Health Organization et al., 2018). Parent–child interaction provides children not only with time spent together, but also with repeated developmental experiences involving language input, emotional responses, behavioral guidance, rule learning, joint attention, and exploratory feedback (Black et al., 2017; Britto et al., 2017; Sameroff, 2009; World Health Organization et al., 2018). When children experience lower-frequency parent–child interaction across consecutive stages, they may have fewer opportunities to receive immediate adult responses, co-regulation, behavioral guidance, and shared task experiences. Recurrent routines may also allow children to practice waiting, shifting attention, following rules, and resolving frustration with immediate feedback. This interpretation is consistent with prior parenting studies linking parenting processes to preschool adjustment and social-emotional competence (Boise & Knoche, 2024; Jeong et al., 2021; Landry et al., 2008; Wang et al., 2023). At the same time, the transactional model implies a plausible reverse pathway: emerging conduct or attention difficulties may disrupt shared routines or make activities harder for caregivers to sustain. The observed associations may therefore reflect reciprocal influence rather than a one-directional effect of interaction frequency.

With regard to specific child health problems, the relatively consistent findings in this study were concentrated in abnormal total emotional and behavioral difficulties, conduct problems, and hyperactivity/inattention, rather than in neurodevelopmental delays. This does not mean that parent–child interaction frequency is unrelated to neurodevelopment, nor should it be interpreted as evidence that emotional and behavioral difficulties are inherently more sensitive. One possibility is that difficulties in emotion regulation, rule following, attention maintenance, and behavioral control are more readily observed and reported by parents during family life and kindergarten adaptation. Delays in communication, motor skills, problem solving, or personal-social ability may require more time or more stable differences before reaching a screening-positive threshold (Croft et al., 2015; Du et al., 2008; Goodman, 1997, 2001; Schonhaut et al., 2013; Squires et al., 2009; Wei et al., 2015). Therefore, the limited evidence related to neurodevelopmental delays in this study should be understood as limited associations observed in the current data, rather than as evidence against long-term neurodevelopmental relevance.

### 4.3. Content-Specific Findings

The findings for parent–child interaction content domains further indicate that different types of family interaction may carry different developmental functions. Storytelling/reading-related activities usually require children to maintain joint attention, understand narrative content, respond to adult questions, and encounter vocabulary, emotional expression, and social rules during interaction (Flack et al., 2018; Hamilton et al., 2016; Mol et al., 2008; Puglisi et al., 2017; Xie et al., 2018). Environmental interaction and learning about nature/animals/plants embed language communication, observation, explanation, and shared exploration in everyday life (Bornstein et al., 2020; Ebert et al., 2020; Ip et al., 2018; Li et al., 2022; Linberg et al., 2020; Purpura et al., 2020; Wu et al., 2020). The associations of these interaction contents with conduct problems, hyperactivity/inattention, and abnormal total emotional and behavioral difficulties suggest that they may be related not only to language and cognitive development but also to children’s everyday behavioral regulation and social adaptation. Related evidence has linked home literacy activities to later early literacy skills (Bergman Deitcher et al., 2024). However, families who sustain these activities may differ in routines, resources, caregiver responsiveness, or child interest; these findings therefore do not isolate an independent causal effect of a particular activity.

This study used both trajectory patterns and cumulative low-level exposure to describe longitudinal exposure to parent–child interaction frequency. These two representations are not redundant. Trajectory patterns reflect the overall form of change in parent–child interaction frequency over time, whereas cumulative low-level exposure reflects the extent to which children repeatedly stayed at a lower interaction level across multiple assessment waves. In analyses of parent–child interaction frequency as a whole, both types of exposure were associated with abnormal total emotional and behavioral difficulties and conduct problems, indicating that both persistently lower and repeatedly lower interaction frequency may be meaningful for research. In the content-domain analyses, trajectory patterns showed more associations than cumulative low-level exposure, suggesting that changes in some interaction contents may add information beyond the number of low-exposure waves. The post hoc analyses also showed that some, but not all, of the longitudinal associations were observed at a single wave. Some trajectory and single-wave subgroups were small, and estimates with wide confidence intervals should be interpreted cautiously (Heinze & Schemper, 2002; Peduzzi et al., 1996; Vittinghoff & McCulloch, 2007).

The activity-level findings provide more detail about what parents and children did together. Lower-frequency reading/picture-book viewing and learning about nature/animals/plants were associated with abnormal total emotional and behavioral difficulties and several specific emotional and behavioral difficulties. Lower-frequency physical activity was associated with screening-positive delay in any neurodevelopmental domain and communication delay. Parent–child physical activity may involve coordinated movement, following directions, sequencing actions, and adjusting behavior in response to adult feedback, which could plausibly relate to communication and motor-related skills. Nevertheless, these activity-level findings arose among many nominal comparisons and may also mark caregiver availability, access to safe space, or child preferences. Because this was an observational study, these associations do not show that any one activity has an independent causal effect. Different activities may instead mark different opportunities for language communication, joint attention, physical participation, and exploratory feedback.

### 4.4. Strengths and Limitations

This study has several strengths. First, the study used three-wave follow-up data, allowing parent–child interaction frequency to be analyzed from a longitudinal exposure perspective rather than being limited to a single time point. Second, the study examined both patterns of change and cumulative low-level exposure, making it possible to distinguish different forms of long-term or repeated lower interaction frequency. Third, the study did not treat parent–child interaction as a single total amount, but further analyzed interaction content domains and specific interactive activities, providing a closer representation of family routines. Fourth, the study included both children’s emotional and behavioral difficulties and neurodevelopmental delays, which helped compare the association patterns of parent–child interaction frequency across different child health problems. Fifth, beyond the main analyses, fully adjusted models and Firth penalized logistic regression sensitivity analyses were conducted, helping to assess whether findings were affected by model complexity, sparse data, or small trajectory subgroups (Heinze & Schemper, 2002; Peduzzi et al., 1996; VanderWeele & Shpitser, 2011; Vittinghoff & McCulloch, 2007).

This study also has limitations. First, parent–child interaction frequency, children’s emotional and behavioral difficulties, and neurodevelopmental delays all came from parent reports, raising the possibility of common-informant, reporting, and social desirability biases. Second, as an observational study, it cannot establish causality. Parenting style, parental mental health, family stress, and child temperament were not measured and may be sources of residual confounding (Sameroff, 2009; VanderWeele & Shpitser, 2011). The results therefore cannot show that increasing a specific activity would reduce child difficulties; intervention studies or stronger causal designs are needed before specific recommendations can be made. Third, low-level exposure was defined within this sample, and the study did not examine whether interaction frequency at the upper end of the distribution offered additional protection. Fourth, no adjustment was made for multiple comparisons, so some nominally significant content-domain, activity-level, or single-wave findings may reflect chance. Fifth, several trajectory, activity-level, and single-wave subgroups contained few children or outcome events, resulting in wide confidence intervals. Large aORs from these models should not be regarded as precise or stable estimates (Heinze & Schemper, 2002; Peduzzi et al., 1996; Vittinghoff & McCulloch, 2007). Accordingly, these findings should guide further research rather than be treated as confirmatory conclusions. Sixth, participation was voluntary and recruitment was limited to participating kindergartens, which may limit generalizability. Finally, follow-up ended during the early period after kindergarten entry and did not cover later academic performance, peer relationships, executive function, or persistent emotional and behavioral difficulties.

### 4.5. Implications and Future Directions

These findings have specific implications at three levels. For measurement, parent–child interaction should be assessed repeatedly and with sufficient content detail to distinguish temporal patterns, cumulative low exposure, and activity mix; a single assessment may capture a temporary disruption rather than a stable routine. For practice, family and early-childhood services may ask whether reported interaction frequency remains low over time and whether participation varies across activities, which may help identify practical barriers such as caregiver time, access to materials or safe space, or difficulty sustaining activities when a child is inattentive or dysregulated. Such discussions should support individualized assistance rather than assign blame or prescribe one activity as a proven remedy. For research, adequately powered, prespecified interventions should test whether feasible changes in routines improve outcomes and whether any effect operates through frequency, responsiveness, content, or their combination (Dong et al., 2023). The present results do not define a minimum interaction dose, establish causal direction, or justify claims that increasing one activity will prevent difficulties.

Future work should combine parent reports with teacher reports, direct observation, or family activity records. Longer follow-up is needed to examine whether longitudinal patterns of parent–child interaction are associated with later school adaptation, executive function, peer relationships, and early academic performance. Studies spanning the full distribution of interaction frequency could assess dose–response patterns, including possible additional protection at very high levels. Intervention studies could then test whether changes in specific interaction content improve children’s behavioral regulation and social adaptation.

These findings indicate that longitudinal exposure patterns of parent–child interaction frequency were more consistently related to emotional and behavioral difficulties than to neurodevelopmental delays among newly enrolled preschool children. Results from the content-domain and specific-activity analyses further show that daily interactive content should be examined alongside overall interaction frequency in future family-based research.

## 5. Conclusions

Based on three-wave follow-up data, this study found that lower longitudinal exposure to parent–child interaction frequency was mainly associated with emotional and behavioral difficulties among newly enrolled preschool children, especially abnormal total emotional and behavioral difficulties, conduct problems, and hyperactivity/inattention. Evidence for neurodevelopmental delays was less consistent and less stable. Future family-based studies should consider changes in interaction frequency over time, repeated low-level exposure, and the developmental opportunities offered by different types of interaction. Longer follow-up, data from multiple informants, and intervention studies or other designs better suited to causal inference are needed.

## Figures and Tables

**Table 1:** Three-wave distribution of parent–child interaction frequency, emotional and behavioral difficulties, ASQ-3 questionnaire versions, and screening-positive neurodevelopmental delay among children with complete repeated measurements (N = 419).

Variable	Baseline Assessment	First Follow-Up	Final Follow-Up	*p* Value Across Waves
Child age, months, median (IQR)	41.77 (39.05–44.84)	44.77 (42.05–47.84)	48.77 (46.05–51.84)	<0.001
Total parent–child interaction frequency score, median (IQR)	32.00 (29.00–35.00)	33.00 (29.00–36.00)	33.00 (30.00–35.00)	0.822
Knowledge-learning interaction frequency score, median (IQR)	8.00 (7.00–9.00)	8.00 (7.00–9.00)	8.00 (7.00–9.00)	0.054
Story-reading interaction frequency score, median (IQR)	6.00 (5.00–7.00)	6.00 (5.00–7.00)	6.00 (5.00–7.00)	<0.001
Physical/cultural interaction frequency score, median (IQR)	10.00 (8.00–11.00)	9.00 (8.00–10.00)	9.00 (8.00–10.00)	<0.001
Environmental interaction frequency score, median (IQR)	9.00 (8.00–10.00)	9.00 (8.00–10.50)	10.00 (8.00–11.00)	<0.001
Low-level overall parent–child interaction frequency, *n* (%)	81 (19.33)	75 (17.90)	96 (22.91)	0.023
Total emotional and behavioral difficulties score, median (IQR)	6.00 (4.00–12.00)	6.00 (4.00–12.00)	7.00 (5.00–10.00)	0.265
Abnormal total emotional and behavioral difficulties, *n* (%)	39 (9.31)	40 (9.55)	38 (9.07)	0.968
ASQ-3 36-month questionnaire version, *n* (%)	103 (24.58)	0 (0.00)	0 (0.00)	--
ASQ-3 42-month questionnaire version, *n* (%)	216 (51.55)	220 (52.51)	69 (16.47)	--
ASQ-3 48-month questionnaire version, *n* (%)	100 (23.87)	199 (47.49)	220 (52.51)	--
ASQ-3 54-month questionnaire version, *n* (%)	0 (0.00)	0 (0.00)	130 (31.03)	--
Screening-positive delay in any neurodevelopmental domain, *n* (%)	40 (9.55)	35 (8.35)	36 (8.59)	0.786

Note. -- indicates that no across-wave statistical test was performed because questionnaire age versions were expected to change with age. IQR, interquartile range.

**Table 2:** Participant characteristics and distribution of abnormal total emotional and behavioral difficulties and screening-positive delay in any neurodevelopmental domain at final follow-up.

Characteristic	Category	*n*	Abnormal Total Emotional and Behavioral Difficulties, *n* (%)	*p* Value for Abnormal Total Emotional and Behavioral Difficulties	Screening-Positive Delay in Any Neurodevelopmental Domain, *n* (%)	*p* Value for Screening-Positive Delay in Any Neurodevelopmental Domain
Overall	All children	419	38 (9.07)	--	36 (8.59)	--
Sex	Female	191	14 (7.33)	0.307	16 (8.38)	1.000
	Male	228	24 (10.53)		20 (8.77)	
Residence	Urban	258	21 (8.14)	0.485	20 (7.75)	0.476
	Rural	161	17 (10.56)		16 (9.94)	
Preschool ownership	Public	261	23 (8.81)	0.861	22 (8.43)	0.860
	Private	158	15 (9.49)		14 (8.86)	
Only child	No	180	17 (9.44)	0.864	18 (10.00)	0.384
	Yes	239	21 (8.79)		18 (7.53)	
Maternal education	Literate but did not complete primary school	6	1 (16.67)	0.759	0 (0.00)	0.627
	Primary school	20	2 (10.00)		1 (5.00)	
	Junior high school	53	6 (11.32)		8 (15.09)	
	Senior high school/technical secondary school	99	10 (10.10)		9 (9.09)	
	Junior college	138	12 (8.70)		10 (7.25)	
	Bachelor’s degree or above	103	7 (6.80)		8 (7.77)	
Annual household income	<10,000 yuan	15	2 (13.33)	0.727	2 (13.33)	0.330
	10,000–29,999 yuan	58	7 (12.07)		8 (13.79)	
	30,000–49,999 yuan	95	9 (9.47)		9 (9.47)	
	50,000–99,999 yuan	138	12 (8.70)		8 (5.80)	
	≥100,000 yuan	113	8 (7.08)		9 (7.96)	
Preterm birth	No	385	34 (8.83)	0.533	30 (7.79)	0.059
	Yes	34	4 (11.76)		6 (17.65)	
Low birth weight	No	398	35 (8.79)	0.424	32 (8.04)	0.095
	Yes	21	3 (14.29)		4 (19.05)	
History of NICU admission	No	393	35 (8.91)	0.720	32 (8.14)	0.264
	Yes	26	3 (11.54)		4 (15.38)	
Ever breastfed	No	47	5 (10.64)	0.599	4 (8.51)	1.000
	Yes	372	33 (8.87)		32 (8.60)	
Maternal pregnancy risk	No	354	30 (8.47)	0.346	27 (7.63)	0.144
	Yes	65	8 (12.31)		9 (13.85)	
Any perinatal risk	No	351	29 (8.26)	0.245	26 (7.41)	0.059
	Yes	68	9 (13.24)		10 (14.71)	

Note. *p* values compare outcome proportions across categories of each participant characteristic. -- indicates not applicable.

## Data Availability

The data are not publicly available due to privacy and ethical restrictions involving child participants. De-identified data may be made available from the corresponding author upon reasonable request and with appropriate approval.

## Supplementary Materials

The following supporting information can be downloaded at: https://www.mdpi.com/article/10.3390/bs16091504/s1, Supplementary Figure S1: participant follow-up flow across the three assessment waves; Supplementary Table S1: complete primary adjusted estimates for overall parent–child interaction frequency trajectories and cumulative low-level exposure in relation to abnormal total emotional and behavioral difficulties, specific emotional and behavioral problems, screening-positive delay in any neurodevelopmental domain, and specific neurodevelopmental delays; Supplementary Table S2: complete primary adjusted estimates for parent–child interaction content-domain trajectories and cumulative low-level exposure in relation to abnormal total emotional and behavioral difficulties, specific emotional and behavioral problems, screening-positive delay in any neurodevelopmental domain, and specific neurodevelopmental delays; Supplementary Table S3: complete primary adjusted estimates for cumulative low-level exposure to specific parent–child interactive activities per additional low-exposure wave in relation to abnormal total emotional and behavioral difficulties, specific emotional and behavioral problems, screening-positive delay in any neurodevelopmental domain, and specific neurodevelopmental delays; Supplementary Table S4: complete fully adjusted estimates for overall and content-domain parent–child interaction frequency trajectories and cumulative low-level exposure in relation to abnormal total emotional and behavioral difficulties, specific emotional and behavioral problems, screening-positive delay in any neurodevelopmental domain, and specific neurodevelopmental delays; Supplementary Table S5: complete Firth penalized estimates for overall and content-domain parent–child interaction frequency trajectories and cumulative low-level exposure in relation to abnormal total emotional and behavioral difficulties, specific emotional and behavioral problems, screening-positive delay in any neurodevelopmental domain, and specific neurodevelopmental delays; Supplementary Table S6: complete adjusted estimates for post hoc analyses of single-wave low-level overall parent–child interaction frequency across emotional-behavioral and neurodevelopmental outcomes; and Supplementary Table S7: complete adjusted estimates for post hoc analyses of single-wave low-level content-domain and activity-level exposures in relation to SDQ and ASQ-3 outcomes.
