# Supplementary material for: Longitudinal Exposure Patterns of Parent–Child Interaction Frequency and Emotional and Behavioral Difficulties and Neurodevelopmental Delays in Preschool Children: A Three-Wave Observational Study
Source: Behav Sci (Basel). 2026 Aug 27;16(9):1504. doi: 10.3390/bs16091504 (PMC13603205; doi:10.3390/bs16091504)
Supplement: Supplementary file 1 [file behavsci-16-01504-s001.zip › behavsci-4337272-supplementary.pdf]

Supplementary Materials

Longitudinal Exposure Patterns of Parent–Child Interaction Frequency and Emotional and Behavioral Difficulties and Neurodevelopmental Delays in Preschool Children: A Three-Wave Observational Study

Contents

Item	Title	Corresponding manuscript section
Supplementary Figure S1	Participant follow-up flow across the three assessment waves.	Methods 2.1
Supplementary Table S1	Complete primary adjusted estimates for overall parent–child interaction frequency trajectories and cumulative low-level exposure in relation to abnormal total emotional and behavioral difficulties, specific emotional and behavioral problems, screening-positive delay in any neurodevelopmental domain, and specific neurodevelopmental delays.	Results 3.2 / Figure 2
Supplementary Table S2	Complete primary adjusted estimates for parent–child interaction content-domain trajectories and cumulative low-level exposure in relation to abnormal total emotional and behavioral difficulties, specific emotional and behavioral problems, screening-positive delay in any neurodevelopmental domain, and specific neurodevelopmental delays.	Results 3.3 / Figure 3
Supplementary Table S3	Complete primary adjusted estimates for cumulative low-level exposure to specific parent–child interactive activities per additional low-exposure wave in relation to abnormal total emotional and behavioral difficulties, specific emotional and behavioral problems, screening-positive delay in any neurodevelopmental domain, and specific neurodevelopmental delays.	Results 3.4 / Figure 4
Supplementary Table S4	Complete fully adjusted estimates for overall and content-domain parent–child interaction frequency trajectories and cumulative low-level exposure in relation to abnormal total emotional and behavioral difficulties, specific emotional and behavioral problems, screening-positive delay in any neurodevelopmental domain, and specific neurodevelopmental delays.	Results 3.5.1
Supplementary Table S5	Complete Firth penalized estimates for overall and content-domain parent–child interaction frequency trajectories and cumulative low-level exposure in relation to abnormal total emotional and behavioral difficulties, specific emotional and behavioral problems, screening-positive delay in any neurodevelopmental domain, and specific neurodevelopmental delays.	Results 3.5.1
Supplementary Table S6	Complete adjusted estimates for post hoc analyses of single-wave low-level overall parent–child interaction frequency across emotional-behavioral and neurodevelopmental outcomes.	Results 3.5.2
Supplementary Table S7	Complete adjusted estimates for post hoc analyses of single-wave low-level content-domain and activity-level exposures in relation to SDQ and ASQ-3 outcomes.	Results 3.5.2

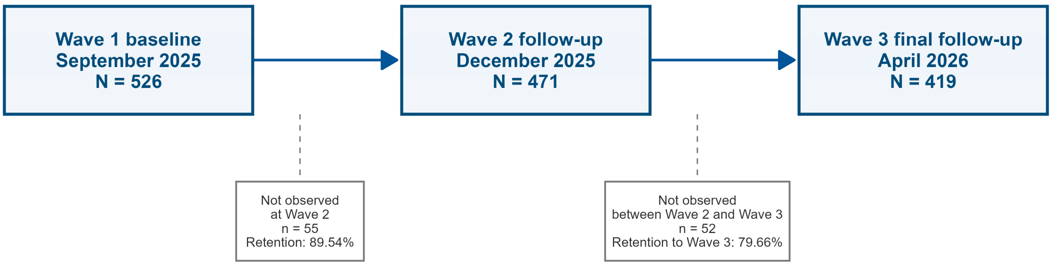

Supplementary Figure S1. Participant follow-up flow across the three assessment waves.

Supplementary Table S1. Complete primary adjusted estimates for overall parent–child interaction frequency trajectories and cumulative low-level exposure in relation to abnormal total emotional and behavioral difficulties, specific emotional and behavioral problems, screening-positive delay in any neurodevelopmental domain, and specific neurodevelopmental delays.

Emotional-behavioral or neurodevelopmental problem group	Specific emotional-behavioral problem or neurodevelopmental delay	Exposure form	Exposure contrast	aOR (95% CI)	*p* value
Emotional and behavioral problems	Abnormal total emotional and behavioral difficulties	Trajectory pattern	Deteriorated trajectory vs persistently normal	2.00 (0.62-6.39)	0.244
			Fluctuating trajectory vs persistently normal	1.70 (0.40-7.19)	0.474
			Improved trajectory vs persistently normal	1.09 (0.22-5.38)	0.916
			Persistently low trajectory vs persistently normal	3.46 (1.01-11.86)	0.049
		Cumulative low-level exposure	Per additional wave of low-level exposure	1.54 (1.02-2.33)	0.041
Neurodevelopmental delay	Screening-positive delay in any neurodevelopmental domain	Trajectory pattern	Deteriorated trajectory vs persistently normal	0.59 (0.15-2.29)	0.444
			Fluctuating trajectory vs persistently normal	0.51 (0.10-2.69)	0.428
			Improved trajectory vs persistently normal	1.44 (0.41-5.09)	0.569
			Persistently low trajectory vs persistently normal	0.55 (0.13-2.30)	0.411
		Cumulative low-level exposure	Per additional wave of low-level exposure	0.89 (0.57-1.40)	0.618
Emotional and behavioral problems	Emotional symptoms in the abnormal range	Trajectory pattern	Deteriorated trajectory vs persistently normal	1.03 (0.29-3.63)	0.962
			Fluctuating trajectory vs persistently normal	0.70 (0.13-3.89)	0.686
			Improved trajectory vs persistently normal	1.10 (0.26-4.62)	0.892
			Persistently low trajectory vs persistently normal	0.69 (0.16-3.07)	0.628

**Emotional-behavioral or neurodevelopmental problem group**	**Specific emotional-behavioral problem or neurodevelopmental delay**	**Exposure form**	**Exposure contrast**	**aOR (95% CI)**	***p* value**
		Cumulative low-level exposure	Per additional wave of low-level exposure	0.97 (0.61-1.55)	0.897
	Conduct problems in the abnormal range	Trajectory pattern	Deteriorated trajectory vs persistently normal	2.93 (0.93-9.27)	0.067
			Fluctuating trajectory vs persistently normal	1.94 (0.44-8.48)	0.381
			Improved trajectory vs persistently normal	2.03 (0.48-8.50)	0.334
			Persistently low trajectory vs persistently normal	3.94 (1.13-13.69)	0.031
		Cumulative low-level exposure	Per additional wave of low-level exposure	1.54 (1.02-2.32)	0.039
	Hyperactivity/inattention in the abnormal range	Trajectory pattern	Deteriorated trajectory vs persistently normal	2.14 (0.57-8.00)	0.259
			Fluctuating trajectory vs persistently normal	1.57 (0.27-9.05)	0.617
			Improved trajectory vs persistently normal	2.64 (0.62-11.35)	0.191
			Persistently low trajectory vs persistently normal	3.26 (0.77-13.70)	0.107
		Cumulative low-level exposure	Per additional wave of low-level exposure	1.50 (0.94-2.41)	0.091
	Peer problems in the abnormal range	Trajectory pattern	Deteriorated trajectory vs persistently normal	1.04 (0.26-4.20)	0.953
			Fluctuating trajectory vs persistently normal	1.48 (0.33-6.65)	0.609
			Improved trajectory vs persistently normal	2.34 (0.64-8.53)	0.196
			Persistently low trajectory vs persistently normal	2.06 (0.54-7.91)	0.290

Emotional-behavioral or neurodevelopmental problem group	Specific emotional-behavioral problem or neurodevelopmental delay	Exposure form	Exposure contrast	aOR (95% CI)	*p* value
		Cumulative low-level exposure	Per additional wave of low-level exposure	1.38 (0.89-2.14)	0.149
	Prosocial behavior difficulties in the abnormal range	Trajectory pattern	Deteriorated trajectory vs persistently normal	1.83 (0.52-6.51)	0.349
			Fluctuating trajectory vs persistently normal	1.99 (0.46-8.69)	0.360
			Improved trajectory vs persistently normal	1.34 (0.27-6.69)	0.725
			Persistently low trajectory vs persistently normal	3.52 (0.96-12.88)	0.057
		Cumulative low-level exposure	Per additional wave of low-level exposure	1.50 (0.97-2.32)	0.065
Neurodevelopmental delay	Communication delay	Trajectory pattern	Deteriorated trajectory vs persistently normal	NA	NA
			Fluctuating trajectory vs persistently normal	0.97 (0.08-11.25)	0.979
			Improved trajectory vs persistently normal	1.22 (0.12-12.84)	0.867
			Persistently low trajectory vs persistently normal	1.58 (0.21-12.07)	0.658
		Cumulative low-level exposure	Per additional wave of low-level exposure	1.15 (0.57-2.31)	0.701
	Gross motor delay	Trajectory pattern	Deteriorated trajectory vs persistently normal	1.33 (0.21-8.31)	0.762
			Fluctuating trajectory vs persistently normal	NA	NA
			Improved trajectory vs persistently normal	2.68 (0.43-16.73)	0.291
			Persistently low trajectory vs persistently normal	0.48 (0.04-6.58)	0.585

**Emotional-behavioral or neurodevelopmental problem group**	**Specific emotional-behavioral problem or neurodevelopmental delay**	**Exposure form**	**Exposure contrast**	**aOR (95% CI)**	***p* value**
		Cumulative low-level exposure	Per additional wave of low-level exposure	1.24 (0.61-2.52)	0.560
	Fine motor delay	Trajectory pattern	Deteriorated trajectory vs persistently normal	0.93 (0.22-3.96)	0.923
			Fluctuating trajectory vs persistently normal	0.77 (0.13-4.58)	0.776
			Improved trajectory vs persistently normal	0.88 (0.16-4.91)	0.887
			Persistently low trajectory vs persistently normal	0.32 (0.05-2.18)	0.245
		Cumulative low-level exposure	Per additional wave of low-level exposure	0.87 (0.50-1.50)	0.609
	Problem-solving delay	Trajectory pattern	Deteriorated trajectory vs persistently normal	NA	NA
			Fluctuating trajectory vs persistently normal	0.42 (0.04-4.18)	0.463
			Improved trajectory vs persistently normal	1.22 (0.22-6.64)	0.822
			Persistently low trajectory vs persistently normal	1.11 (0.21-5.78)	0.901
		Cumulative low-level exposure	Per additional wave of low-level exposure	0.98 (0.55-1.74)	0.940
	Personal-social delay	Trajectory pattern	Deteriorated trajectory vs persistently normal	0.50 (0.05-4.80)	0.552
			Fluctuating trajectory vs persistently normal	NA	NA
			Improved trajectory vs persistently normal	2.01 (0.35-11.64)	0.434
			Persistently low trajectory vs persistently normal	0.37 (0.03-4.55)	0.436

**Emotional-behavioral or neurodevelopmental problem group**	**Specific emotional-behavioral problem or neurodevelopmental delay**	**Exposure form**	**Exposure contrast**	**aOR (95% CI)**	***p* value**
		Cumulative low-level exposure	Per additional wave of low-level exposure	0.74 (0.35-1.59)	0.439

Note. Values are adjusted odds ratios from primary adjusted logistic regression models. Overall parent–child interaction frequency is the exposure for all estimates in this table. NA indicates that an estimate was not reported because of an unstable model estimate.

Supplementary Table S2. Complete primary adjusted estimates for parent–child interaction content-domain trajectories and cumulative low-level exposure in relation to abnormal total emotional and behavioral difficulties, specific emotional and behavioral problems, screening-positive delay in any neurodevelopmental domain, and specific neurodevelopmental delays.

Emotional-behavioral or neurodevelopmental problem group	Specific emotional-behavioral problem or neurodevelopmental delay	Parent–child interaction measure	Exposure form	Exposure contrast	aOR (95% CI)	*p* value
Emotional and behavioral problems	Abnormal total emotional and behavioral difficulties	Knowledge-learning interaction frequency	Trajectory pattern	Deteriorated trajectory vs persistently normal	1.14 (0.34-3.82)	0.829
				Fluctuating trajectory vs persistently normal	0.38 (0.08-1.87)	0.236
				Improved trajectory vs persistently normal	0.64 (0.20-2.05)	0.458
				Persistently low trajectory vs persistently normal	1.86 (0.47-7.34)	0.374
			Cumulative low-level exposure	Per additional wave of low-level exposure	1.13 (0.72-1.76)	0.595
		Story-reading interaction frequency	Trajectory pattern	Deteriorated trajectory vs persistently normal	0.96 (0.20-4.71)	0.960
				Fluctuating trajectory vs persistently normal	3.12 (1.04-9.32)	0.042
				Improved trajectory vs persistently normal	0.64 (0.17-2.37)	0.502
				Persistently low trajectory vs persistently normal	5.85 (1.20-28.60)	0.029
			Cumulative low-level exposure	Per additional wave of low-level exposure	1.44 (0.91-2.29)	0.119
		Physical/cultural interaction frequency	Trajectory pattern	Deteriorated trajectory vs persistently normal	0.92 (0.33-2.55)	0.872
				Fluctuating trajectory vs persistently normal	1.06 (0.33-3.41)	0.924
				Improved trajectory vs persistently normal	0.44 (0.05-3.60)	0.446

Emotional-behavioral or neurodevelopmental problem group	Specific emotional-behavioral problem or neurodevelopmental delay	Parent–child interaction measure	Exposure form	Exposure contrast	aOR (95% CI)	*p* value
				Persistently low trajectory vs persistently normal	2.28 (0.52-9.98)	0.275
			Cumulative low-level exposure	Per additional wave of low-level exposure	1.16 (0.75-1.82)	0.503
		Environmental interaction frequency	Trajectory pattern	Deteriorated trajectory vs persistently normal	1.62 (0.40-6.60)	0.501
				Fluctuating trajectory vs persistently normal	1.56 (0.45-5.44)	0.487
				Improved trajectory vs persistently normal	1.32 (0.40-4.31)	0.646
				Persistently low trajectory vs persistently normal	10.59 (1.89-59.39)	0.007
			Cumulative low-level exposure	Per additional wave of low-level exposure	1.54 (0.96-2.47)	0.074
Neurodevelopmental delay	Screening-positive delay in any neurodevelopmental domain	Knowledge-learning interaction frequency	Trajectory pattern	Deteriorated trajectory vs persistently normal	0.81 (0.21-3.15)	0.763
				Fluctuating trajectory vs persistently normal	1.03 (0.32-3.29)	0.962
				Improved trajectory vs persistently normal	0.62 (0.19-2.00)	0.425
				Persistently low trajectory vs persistently normal	0.33 (0.04-2.86)	0.313
			Cumulative low-level exposure	Per additional wave of low-level exposure	0.69 (0.42-1.16)	0.163
		Story-reading interaction frequency	Trajectory pattern	Deteriorated trajectory vs persistently normal	1.21 (0.31-4.82)	0.783
				Fluctuating trajectory vs persistently normal	1.16 (0.30-4.45)	0.831

Emotional-behavioral or neurodevelopmental problem group	Specific emotional-behavioral problem or neurodevelopmental delay	Parent–child interaction measure	Exposure form	Exposure contrast	aOR (95% CI)	*p* value
				Improved trajectory vs persistently normal	0.93 (0.31-2.82)	0.895
				Persistently low trajectory vs persistently normal	NA	NA
			Cumulative low-level exposure	Per additional wave of low-level exposure	0.84 (0.48-1.46)	0.538
		Physical/cultural interaction frequency	Trajectory pattern	Deteriorated trajectory vs persistently normal	1.12 (0.42-3.01)	0.824
				Fluctuating trajectory vs persistently normal	0.37 (0.07-1.85)	0.227
				Improved trajectory vs persistently normal	0.43 (0.05-3.56)	0.436
				Persistently low trajectory vs persistently normal	2.36 (0.53-10.53)	0.259
			Cumulative low-level exposure	Per additional wave of low-level exposure	1.00 (0.61-1.61)	0.986
		Environmental interaction frequency	Trajectory pattern	Deteriorated trajectory vs persistently normal	0.32 (0.04-2.71)	0.298
				Fluctuating trajectory vs persistently normal	1.16 (0.37-3.66)	0.795
				Improved trajectory vs persistently normal	0.45 (0.10-2.09)	0.309
				Persistently low trajectory vs persistently normal	1.30 (0.13-12.57)	0.822
			Cumulative low-level exposure	Per additional wave of low-level exposure	0.74 (0.41-1.34)	0.324
	Emotional symptoms in the abnormal range	Knowledge-learning interaction frequency	Trajectory pattern	Deteriorated trajectory vs persistently normal	0.75 (0.19-2.97)	0.684

Emotional-behavioral or neurodevelopmental problem group	Specific emotional-behavioral problem or neurodevelopmental delay	Parent–child interaction measure	Exposure form	Exposure contrast	aOR (95% CI)	*p* value
Emotional and behavioral problems				Fluctuating trajectory vs persistently normal	0.14 (0.02-1.26)	0.079
				Improved trajectory vs persistently normal	0.58 (0.17-1.90)	0.366
				Persistently low trajectory vs persistently normal	0.75 (0.13-4.20)	0.744
			Cumulative low-level exposure	Per additional wave of low-level exposure	0.80 (0.47-1.35)	0.397
		Story-reading interaction frequency	Trajectory pattern	Deteriorated trajectory vs persistently normal	NA	NA
				Fluctuating trajectory vs persistently normal	1.87 (0.55-6.34)	0.316
				Improved trajectory vs persistently normal	0.31 (0.07-1.48)	0.143
				Persistently low trajectory vs persistently normal	1.08 (0.11-10.18)	0.949
			Cumulative low-level exposure	Per additional wave of low-level exposure	0.78 (0.43-1.44)	0.429
		Physical/cultural interaction frequency	Trajectory pattern	Deteriorated trajectory vs persistently normal	0.36 (0.10-1.37)	0.135
				Fluctuating trajectory vs persistently normal	0.61 (0.17-2.22)	0.449
				Improved trajectory vs persistently normal	0.37 (0.04-3.14)	0.360
				Persistently low trajectory vs persistently normal	1.25 (0.22-7.19)	0.799
			Cumulative low-level exposure	Per additional wave of low-level exposure	0.87 (0.51-1.48)	0.597

**Emotional-behavioral or neurodevelopmental problem group**	**Specific emotional-behavioral problem or neurodevelopmental delay**	**Parent–child interaction measure**	**Exposure form**	**Exposure contrast**	**aOR (95% CI)**	***p* value**
		Environmental interaction frequency	Trajectory pattern	Deteriorated trajectory vs persistently normal	1.38 (0.31-6.12)	0.674
				Fluctuating trajectory vs persistently normal	0.74 (0.14-3.77)	0.715
				Improved trajectory vs persistently normal	1.41 (0.41-4.85)	0.582
				Persistently low trajectory vs persistently normal	6.31 (0.91-44.00)	0.063
			Cumulative low-level exposure	Per additional wave of low-level exposure	1.25 (0.73-2.15)	0.414
	Conduct problems in the abnormal range	Knowledge-learning interaction frequency	Trajectory pattern	Deteriorated trajectory vs persistently normal	0.82 (0.21-3.25)	0.779
				Fluctuating trajectory vs persistently normal	0.33 (0.07-1.67)	0.180
				Improved trajectory vs persistently normal	0.64 (0.19-2.09)	0.458
				Persistently low trajectory vs persistently normal	2.69 (0.75-9.64)	0.127
			Cumulative low-level exposure	Per additional wave of low-level exposure	1.30 (0.85-2.00)	0.230
		Story-reading interaction frequency	Trajectory pattern	Deteriorated trajectory vs persistently normal	0.82 (0.16-4.17)	0.814
				Fluctuating trajectory vs persistently normal	1.64 (0.46-5.77)	0.444
				Improved trajectory vs persistently normal	1.15 (0.40-3.32)	0.792
				Persistently low trajectory vs persistently normal	5.33 (1.08-26.25)	0.040

Emotional-behavioral or neurodevelopmental problem group	Specific emotional-behavioral problem or neurodevelopmental delay	Parent–child interaction measure	Exposure form	Exposure contrast	aOR (95% CI)	*p* value
			Cumulative low-level exposure	Per additional wave of low-level exposure	1.40 (0.88-2.22)	0.152
		Physical/cultural interaction frequency	Trajectory pattern	Deteriorated trajectory vs persistently normal	0.80 (0.28-2.28)	0.671
				Fluctuating trajectory vs persistently normal	0.64 (0.18-2.33)	0.498
				Improved trajectory vs persistently normal	0.70 (0.14-3.54)	0.671
				Persistently low trajectory vs persistently normal	1.90 (0.42-8.71)	0.408
			Cumulative low-level exposure	Per additional wave of low-level exposure	0.99 (0.62-1.57)	0.954
		Environmental interaction frequency	Trajectory pattern	Deteriorated trajectory vs persistently normal	0.65 (0.13-3.35)	0.610
				Fluctuating trajectory vs persistently normal	1.09 (0.31-3.85)	0.898
				Improved trajectory vs persistently normal	1.38 (0.46-4.19)	0.567
				Persistently low trajectory vs persistently normal	6.74 (1.22-37.23)	0.029
			Cumulative low-level exposure	Per additional wave of low-level exposure	1.46 (0.92-2.32)	0.112
	Hyperactivity/inattention in the abnormal range	Knowledge-learning interaction frequency	Trajectory pattern	Deteriorated trajectory vs persistently normal	1.04 (0.26-4.19)	0.951
				Fluctuating trajectory vs persistently normal	0.21 (0.02-1.88)	0.164
				Improved trajectory vs persistently normal	1.11 (0.36-3.43)	0.856

Emotional-behavioral or neurodevelopmental problem group	Specific emotional-behavioral problem or neurodevelopmental delay	Parent–child interaction measure	Exposure form	Exposure contrast	aOR (95% CI)	*p* value
				Persistently low trajectory vs persistently normal	1.01 (0.18-5.69)	0.992
			Cumulative low-level exposure	Per additional wave of low-level exposure	1.05 (0.63-1.76)	0.846
		Story-reading interaction frequency	Trajectory pattern	Deteriorated trajectory vs persistently normal	0.65 (0.07-5.68)	0.699
				Fluctuating trajectory vs persistently normal	2.77 (0.75-10.21)	0.126
				Improved trajectory vs persistently normal	1.35 (0.38-4.71)	0.642
				Persistently low trajectory vs persistently normal	9.50 (1.83-49.40)	0.007
			Cumulative low-level exposure	Per additional wave of low-level exposure	1.54 (0.92-2.57)	0.102
		Physical/cultural interaction frequency	Trajectory pattern	Deteriorated trajectory vs persistently normal	1.70 (0.56-5.17)	0.354
				Fluctuating trajectory vs persistently normal	1.43 (0.37-5.53)	0.601
				Improved trajectory vs persistently normal	NA	NA
				Persistently low trajectory vs persistently normal	6.26 (1.41-27.86)	0.016
			Cumulative low-level exposure	Per additional wave of low-level exposure	1.54 (0.94-2.51)	0.086
		Environmental interaction frequency	Trajectory pattern	Deteriorated trajectory vs persistently normal	1.78 (0.40-7.93)	0.451
				Fluctuating trajectory vs persistently normal	0.90 (0.17-4.70)	0.905

Emotional-behavioral or neurodevelopmental problem group	Specific emotional-behavioral problem or neurodevelopmental delay	Parent–child interaction measure	Exposure form	Exposure contrast	aOR (95% CI)	*p* value
				Improved trajectory vs persistently normal	0.80 (0.16-3.95)	0.788
				Persistently low trajectory vs persistently normal	12.19 (2.00-74.39)	0.007
			Cumulative low-level exposure	Per additional wave of low-level exposure	1.62 (0.95-2.75)	0.075
	Peer problems in the abnormal range	Knowledge-learning interaction frequency	Trajectory pattern	Deteriorated trajectory vs persistently normal	1.25 (0.36-4.31)	0.723
				Fluctuating trajectory vs persistently normal	0.19 (0.02-1.61)	0.128
				Improved trajectory vs persistently normal	0.73 (0.22-2.41)	0.609
				Persistently low trajectory vs persistently normal	2.15 (0.52-8.97)	0.294
			Cumulative low-level exposure	Per additional wave of low-level exposure	1.07 (0.67-1.73)	0.770
		Story-reading interaction frequency	Trajectory pattern	Deteriorated trajectory vs persistently normal	0.77 (0.15-3.84)	0.749
				Fluctuating trajectory vs persistently normal	1.58 (0.45-5.54)	0.477
				Improved trajectory vs persistently normal	0.73 (0.22-2.40)	0.607
				Persistently low trajectory vs persistently normal	NA	NA
			Cumulative low-level exposure	Per additional wave of low-level exposure	0.81 (0.46-1.44)	0.476
		Physical/cultural interaction frequency	Trajectory pattern	Deteriorated trajectory vs persistently normal	0.41 (0.11-1.56)	0.192

Emotional-behavioral or neurodevelopmental problem group	Specific emotional-behavioral problem or neurodevelopmental delay	Parent–child interaction measure	Exposure form	Exposure contrast	aOR (95% CI)	*p* value
				Fluctuating trajectory vs persistently normal	1.42 (0.48-4.23)	0.527
				Improved trajectory vs persistently normal	0.45 (0.05-3.69)	0.456
				Persistently low trajectory vs persistently normal	1.23 (0.22-6.76)	0.812
			Cumulative low-level exposure	Per additional wave of low-level exposure	0.94 (0.57-1.53)	0.795
		Environmental interaction frequency	Trajectory pattern	Deteriorated trajectory vs persistently normal	1.06 (0.20-5.55)	0.947
				Fluctuating trajectory vs persistently normal	1.92 (0.53-6.95)	0.321
				Improved trajectory vs persistently normal	1.94 (0.62-6.02)	0.253
				Persistently low trajectory vs persistently normal	5.89 (0.88-39.52)	0.068
			Cumulative low-level exposure	Per additional wave of low-level exposure	1.44 (0.87-2.37)	0.156
	Prosocial behavior difficulties in the abnormal range	Knowledge-learning interaction frequency	Trajectory pattern	Deteriorated trajectory vs persistently normal	0.68 (0.14-3.28)	0.635
				Fluctuating trajectory vs persistently normal	0.24 (0.03-2.03)	0.193
				Improved trajectory vs persistently normal	1.07 (0.36-3.18)	0.910
				Persistently low trajectory vs persistently normal	3.29 (0.85-12.64)	0.083
			Cumulative low-level exposure	Per additional wave of low-level exposure	1.51 (0.96-2.38)	0.073

Emotional-behavioral or neurodevelopmental problem group	Specific emotional-behavioral problem or neurodevelopmental delay	Parent–child interaction measure	Exposure form	Exposure contrast	aOR (95% CI)	*p* value
		Story-reading interaction frequency	Trajectory pattern	Deteriorated trajectory vs persistently normal	0.95 (0.19-4.69)	0.952
				Fluctuating trajectory vs persistently normal	0.85 (0.18-4.10)	0.839
				Improved trajectory vs persistently normal	1.65 (0.61-4.49)	0.328
				Persistently low trajectory vs persistently normal	NA	NA
			Cumulative low-level exposure	Per additional wave of low-level exposure	1.16 (0.70-1.94)	0.565
		Physical/cultural interaction frequency	Trajectory pattern	Deteriorated trajectory vs persistently normal	0.91 (0.32-2.56)	0.851
				Fluctuating trajectory vs persistently normal	0.59 (0.15-2.35)	0.452
				Improved trajectory vs persistently normal	0.43 (0.05-3.53)	0.432
				Persistently low trajectory vs persistently normal	1.25 (0.23-6.77)	0.795
			Cumulative low-level exposure	Per additional wave of low-level exposure	0.97 (0.59-1.58)	0.889
		Environmental interaction frequency	Trajectory pattern	Deteriorated trajectory vs persistently normal	1.12 (0.28-4.53)	0.869
				Fluctuating trajectory vs persistently normal	0.51 (0.11-2.48)	0.406
				Improved trajectory vs persistently normal	0.72 (0.20-2.67)	0.625
				Persistently low trajectory vs persistently normal	NA	NA

**Emotional-behavioral or neurodevelopmental problem group**	**Specific emotional-behavioral problem or neurodevelopmental delay**	**Parent–child interaction measure**	**Exposure form**	**Exposure contrast**	**aOR (95% CI)**	***p* value**
			Cumulative low-level exposure	Per additional wave of low-level exposure	0.82 (0.46-1.48)	0.515
Neurodevelopmental delay	Communication delay	Knowledge-learning interaction frequency	Trajectory pattern	Deteriorated trajectory vs persistently normal	0.94 (0.09-9.76)	0.962
				Fluctuating trajectory vs persistently normal	1.90 (0.34-10.71)	0.465
				Improved trajectory vs persistently normal	0.97 (0.17-5.60)	0.976
				Persistently low trajectory vs persistently normal	1.00 (0.09-11.26)	0.999
			Cumulative low-level exposure	Per additional wave of low-level exposure	0.93 (0.46-1.90)	0.843
		Story-reading interaction frequency	Trajectory pattern	Deteriorated trajectory vs persistently normal	0.77 (0.08-7.89)	0.829
				Fluctuating trajectory vs persistently normal	0.81 (0.09-7.72)	0.855
				Improved trajectory vs persistently normal	1.16 (0.24-5.67)	0.853
				Persistently low trajectory vs persistently normal	NA	NA
			Cumulative low-level exposure	Per additional wave of low-level exposure	0.88 (0.39-2.01)	0.766
		Physical/cultural interaction frequency	Trajectory pattern	Deteriorated trajectory vs persistently normal	0.72 (0.12-4.35)	0.716
				Fluctuating trajectory vs persistently normal	0.35 (0.03-3.72)	0.385
				Improved trajectory vs persistently normal	1.25 (0.12-12.88)	0.852

Emotional-behavioral or neurodevelopmental problem group	Specific emotional-behavioral problem or neurodevelopmental delay	Parent–child interaction measure	Exposure form	Exposure contrast	aOR (95% CI)	*p* value
				Persistently low trajectory vs persistently normal	1.95 (0.17-22.46)	0.592
			Cumulative low-level exposure	Per additional wave of low-level exposure	0.92 (0.41-2.03)	0.834
		Environmental interaction frequency	Trajectory pattern	Deteriorated trajectory vs persistently normal	0.65 (0.06-6.71)	0.721
				Fluctuating trajectory vs persistently normal	0.72 (0.12-4.21)	0.714
				Improved trajectory vs persistently normal	0.55 (0.06-5.10)	0.599
				Persistently low trajectory vs persistently normal	NA	NA
			Cumulative low-level exposure	Per additional wave of low-level exposure	0.62 (0.24-1.61)	0.325
	Gross motor delay	Knowledge-learning interaction frequency	Trajectory pattern	Deteriorated trajectory vs persistently normal	0.93 (0.10-8.83)	0.952
				Fluctuating trajectory vs persistently normal	0.60 (0.06-6.35)	0.673
				Improved trajectory vs persistently normal	0.48 (0.05-4.43)	0.516
				Persistently low trajectory vs persistently normal	1.36 (0.12-15.43)	0.804
			Cumulative low-level exposure	Per additional wave of low-level exposure	0.92 (0.41-2.06)	0.838
		Story-reading interaction frequency	Trajectory pattern	Deteriorated trajectory vs persistently normal	1.53 (0.14-16.23)	0.726
				Fluctuating trajectory vs persistently normal	4.44 (0.88-22.45)	0.072

Emotional-behavioral or neurodevelopmental problem group	Specific emotional-behavioral problem or neurodevelopmental delay	Parent–child interaction measure	Exposure form	Exposure contrast	aOR (95% CI)	*p* value
				Improved trajectory vs persistently normal	NA	NA
				Persistently low trajectory vs persistently normal	NA	NA
			Cumulative low-level exposure	Per additional wave of low-level exposure	1.17 (0.50-2.72)	0.723
		Physical/cultural interaction frequency	Trajectory pattern	Deteriorated trajectory vs persistently normal	1.53 (0.31-7.56)	0.599
				Fluctuating trajectory vs persistently normal	0.72 (0.07-7.84)	0.787
				Improved trajectory vs persistently normal	NA	NA
				Persistently low trajectory vs persistently normal	2.25 (0.18-27.51)	0.527
			Cumulative low-level exposure	Per additional wave of low-level exposure	1.10 (0.49-2.47)	0.820
		Environmental interaction frequency	Trajectory pattern	Deteriorated trajectory vs persistently normal	NA	NA
				Fluctuating trajectory vs persistently normal	1.99 (0.31-12.66)	0.467
				Improved trajectory vs persistently normal	0.77 (0.08-7.15)	0.819
				Persistently low trajectory vs persistently normal	NA	NA
			Cumulative low-level exposure	Per additional wave of low-level exposure	0.58 (0.19-1.76)	0.341
	Fine motor delay	Knowledge-learning interaction frequency	Trajectory pattern	Deteriorated trajectory vs persistently normal	0.90 (0.17-4.70)	0.899

Emotional-behavioral or neurodevelopmental problem group	Specific emotional-behavioral problem or neurodevelopmental delay	Parent–child interaction measure	Exposure form	Exposure contrast	aOR (95% CI)	*p* value
				Fluctuating trajectory vs persistently normal	1.71 (0.47-6.20)	0.412
				Improved trajectory vs persistently normal	0.51 (0.10-2.49)	0.403
				Persistently low trajectory vs persistently normal	NA	NA
			Cumulative low-level exposure	Per additional wave of low-level exposure	0.61 (0.32-1.16)	0.133
		Story-reading interaction frequency	Trajectory pattern	Deteriorated trajectory vs persistently normal	0.96 (0.18-5.21)	0.965
				Fluctuating trajectory vs persistently normal	1.50 (0.36-6.33)	0.582
				Improved trajectory vs persistently normal	0.20 (0.02-1.70)	0.141
				Persistently low trajectory vs persistently normal	NA	NA
			Cumulative low-level exposure	Per additional wave of low-level exposure	0.78 (0.39-1.56)	0.478
		Physical/cultural interaction frequency	Trajectory pattern	Deteriorated trajectory vs persistently normal	1.52 (0.49-4.70)	0.468
				Fluctuating trajectory vs persistently normal	0.52 (0.09-2.87)	0.453
				Improved trajectory vs persistently normal	0.65 (0.07-5.71)	0.694
				Persistently low trajectory vs persistently normal	NA	NA
			Cumulative low-level exposure	Per additional wave of low-level exposure	0.73 (0.38-1.41)	0.355

**Emotional-behavioral or neurodevelopmental problem group**	**Specific emotional-behavioral problem or neurodevelopmental delay**	**Parent–child interaction measure**	**Exposure form**	**Exposure contrast**	**aOR (95% CI)**	***p* value**
		Environmental interaction frequency	Trajectory pattern	Deteriorated trajectory vs persistently normal	0.49 (0.05-4.48)	0.527
				Fluctuating trajectory vs persistently normal	1.56 (0.42-5.79)	0.509
				Improved trajectory vs persistently normal	0.80 (0.16-3.95)	0.783
				Persistently low trajectory vs persistently normal	NA	NA
			Cumulative low-level exposure	Per additional wave of low-level exposure	0.63 (0.29-1.35)	0.232
	Problem-solving delay	Knowledge-learning interaction frequency	Trajectory pattern	Deteriorated trajectory vs persistently normal	0.38 (0.04-3.51)	0.396
				Fluctuating trajectory vs persistently normal	0.62 (0.12-3.38)	0.585
				Improved trajectory vs persistently normal	0.82 (0.20-3.36)	0.786
				Persistently low trajectory vs persistently normal	0.49 (0.05-4.87)	0.539
			Cumulative low-level exposure	Per additional wave of low-level exposure	0.83 (0.44-1.58)	0.576
		Story-reading interaction frequency	Trajectory pattern	Deteriorated trajectory vs persistently normal	1.50 (0.28-8.12)	0.641
				Fluctuating trajectory vs persistently normal	0.66 (0.08-5.78)	0.711
				Improved trajectory vs persistently normal	1.02 (0.25-4.21)	0.981
				Persistently low trajectory vs persistently normal	NA	NA

Emotional-behavioral or neurodevelopmental problem group	Specific emotional-behavioral problem or neurodevelopmental delay	Parent–child interaction measure	Exposure form	Exposure contrast	aOR (95% CI)	*p* value
			Cumulative low-level exposure	Per additional wave of low-level exposure	0.75 (0.35-1.61)	0.463
		Physical/cultural interaction frequency	Trajectory pattern	Deteriorated trajectory vs persistently normal	0.78 (0.19-3.18)	0.730
				Fluctuating trajectory vs persistently normal	0.35 (0.04-3.14)	0.345
				Improved trajectory vs persistently normal	0.82 (0.09-7.12)	0.855
				Persistently low trajectory vs persistently normal	2.49 (0.41-15.16)	0.321
			Cumulative low-level exposure	Per additional wave of low-level exposure	1.01 (0.54-1.87)	0.980
		Environmental interaction frequency	Trajectory pattern	Deteriorated trajectory vs persistently normal	0.76 (0.08-6.88)	0.807
				Fluctuating trajectory vs persistently normal	1.42 (0.33-6.20)	0.637
				Improved trajectory vs persistently normal	0.46 (0.05-3.85)	0.472
				Persistently low trajectory vs persistently normal	3.03 (0.27-33.51)	0.366
			Cumulative low-level exposure	Per additional wave of low-level exposure	1.01 (0.50-2.03)	0.979
	Personal-social delay	Knowledge-learning interaction frequency	Trajectory pattern	Deteriorated trajectory vs persistently normal	0.82 (0.09-7.44)	0.859
				Fluctuating trajectory vs persistently normal	1.19 (0.20-6.96)	0.850
				Improved trajectory vs persistently normal	0.45 (0.05-3.86)	0.466

**Emotional-behavioral or neurodevelopmental problem group**	**Specific emotional-behavioral problem or neurodevelopmental delay**	**Parent–child interaction measure**	**Exposure form**	**Exposure contrast**	**aOR (95% CI)**	***p* value**
				Persistently low trajectory vs persistently normal	NA	NA
			Cumulative low-level exposure	Per additional wave of low-level exposure	0.62 (0.26-1.51)	0.294
		Story-reading interaction frequency	Trajectory pattern	Deteriorated trajectory vs persistently normal	3.46 (0.57-20.91)	0.176
				Fluctuating trajectory vs persistently normal	1.49 (0.16-13.65)	0.727
				Improved trajectory vs persistently normal	1.52 (0.27-8.60)	0.637
				Persistently low trajectory vs persistently normal	NA	NA
			Cumulative low-level exposure	Per additional wave of low-level exposure	1.31 (0.60-2.85)	0.503
		Physical/cultural interaction frequency	Trajectory pattern	Deteriorated trajectory vs persistently normal	2.18 (0.47-10.14)	0.320
				Fluctuating trajectory vs persistently normal	1.09 (0.10-11.36)	0.945
				Improved trajectory vs persistently normal	NA	NA
				Persistently low trajectory vs persistently normal	8.33 (1.07-64.93)	0.043
			Cumulative low-level exposure	Per additional wave of low-level exposure	1.49 (0.73-3.05)	0.274
		Environmental interaction frequency	Trajectory pattern	Deteriorated trajectory vs persistently normal	NA	NA
				Fluctuating trajectory vs persistently normal	NA	NA

Emotional-behavioral or neurodevelopmental problem group	Specific emotional-behavioral problem or neurodevelopmental delay	Parent–child interaction measure	Exposure form	Exposure contrast	aOR (95% CI)	*p* value
				Improved trajectory vs persistently normal	0.58 (0.07-5.01)	0.622
				Persistently low trajectory vs persistently normal	3.36 (0.29-39.33)	0.334
			Cumulative low-level exposure	Per additional wave of low-level exposure	0.76 (0.29-2.01)	0.584

Note. Values are adjusted odds ratios from primary adjusted logistic regression models. NA indicates that an estimate was not reported because of an unstable model estimate.

Supplementary Table S3. Complete primary adjusted estimates for cumulative low-level exposure to specific parent–child interactive activities per additional low-exposure wave in relation to abnormal total emotional and behavioral difficulties, specific emotional and behavioral problems, screening-positive delay in any neurodevelopmental domain, and specific neurodevelopmental delays.

Emotional-behavioral or neurodevelopmental problem group	Specific emotional-behavioral problem or neurodevelopmental delay	Parent–child interactive activity	aOR (95% CI)	*p* value
Emotional and behavioral problems	Abnormal total emotional and behavioral difficulties	Reading picture books	2.24 (1.05-4.75)	0.036
		Teaching number concepts	1.25 (0.76-2.08)	0.380
		Drawing or painting	0.84 (0.48-1.47)	0.539
		Cognitive games	1.06 (0.57-1.96)	0.859
		Learning characters in daily life	1.02 (0.64-1.62)	0.927
		Songs, poems, or nursery rhymes	1.35 (0.72-2.55)	0.353
		Storytelling	1.59 (1.00-2.54)	0.050
		Handicrafts	0.77 (0.35-1.70)	0.524
		Physical activity	0.95 (0.58-1.57)	0.848
		Self-care skills	0.69 (0.33-1.42)	0.314
		Discussing surrounding events	0.90 (0.55-1.48)	0.684
		Learning about nature, animals, or plants	1.71 (1.02-2.88)	0.043
Neurodevelopmental delay	Screening-positive delay in any neurodevelopmental domain	Reading picture books	1.18 (0.48-2.86)	0.718
		Teaching number concepts	0.95 (0.54-1.65)	0.845
		Drawing or painting	0.79 (0.44-1.42)	0.437
		Cognitive games	1.09 (0.58-2.05)	0.779
		Learning characters in daily life	0.75 (0.45-1.26)	0.280
		Songs, poems, or nursery rhymes	1.12 (0.56-2.24)	0.751
		Storytelling	0.83 (0.48-1.45)	0.515
		Handicrafts	0.82 (0.37-1.81)	0.628
		Physical activity	1.64 (1.03-2.62)	0.039

**Emotional-behavioral or neurodevelopmental problem group**	**Specific emotional-behavioral problem or neurodevelopmental delay**	**Parent–child interactive activity**	**aOR (95% CI)**	***p* value**
		Self-care skills	0.89 (0.45-1.77)	0.740
		Discussing surrounding events	0.99 (0.60-1.63)	0.964
		Learning about nature, animals, or plants	0.77 (0.37-1.62)	0.493
Emotional and behavioral problems	Emotional symptoms in the abnormal range	Reading picture books	1.24 (0.47-3.30)	0.664
		Teaching number concepts	1.36 (0.78-2.37)	0.278
		Drawing or painting	0.84 (0.45-1.58)	0.597
		Cognitive games	0.78 (0.37-1.64)	0.507
		Learning characters in daily life	0.87 (0.52-1.46)	0.608
		Songs, poems, or nursery rhymes	1.20 (0.60-2.41)	0.604
		Storytelling	0.95 (0.55-1.64)	0.849
		Handicrafts	0.41 (0.13-1.32)	0.135
		Physical activity	0.97 (0.56-1.67)	0.909
		Self-care skills	0.76 (0.36-1.62)	0.483
		Discussing surrounding events	0.71 (0.39-1.28)	0.254
		Learning about nature, animals, or plants	1.13 (0.57-2.22)	0.723
	Conduct problems in the abnormal range	Reading picture books	1.96 (0.92-4.17)	0.080
		Teaching number concepts	1.27 (0.76-2.11)	0.362
		Drawing or painting	0.97 (0.57-1.63)	0.904
		Cognitive games	1.45 (0.84-2.51)	0.187
		Learning characters in daily life	1.19 (0.77-1.85)	0.437
		Songs, poems, or nursery rhymes	1.34 (0.71-2.55)	0.371
		Storytelling	1.18 (0.73-1.91)	0.504
		Handicrafts	0.76 (0.34-1.69)	0.499

**Emotional-behavioral or neurodevelopmental problem group**	**Specific emotional-behavioral problem or neurodevelopmental delay**	**Parent–child interactive activity**	**aOR (95% CI)**	***p* value**
		Physical activity	0.87 (0.52-1.46)	0.590
		Self-care skills	0.82 (0.41-1.61)	0.554
		Discussing surrounding events	0.75 (0.45-1.27)	0.283
		Learning about nature, animals, or plants	1.77 (1.06-2.97)	0.030
	Hyperactivity/inattention in the abnormal range	Reading picture books	2.36 (1.03-5.39)	0.041
		Teaching number concepts	0.96 (0.52-1.80)	0.907
		Drawing or painting	0.72 (0.36-1.43)	0.349
		Cognitive games	1.22 (0.64-2.34)	0.550
		Learning characters in daily life	0.99 (0.59-1.67)	0.983
		Songs, poems, or nursery rhymes	1.97 (0.99-3.90)	0.053
		Storytelling	1.32 (0.77-2.26)	0.306
		Handicrafts	0.76 (0.31-1.90)	0.560
		Physical activity	0.99 (0.56-1.76)	0.981
		Self-care skills	0.73 (0.33-1.62)	0.444
		Discussing surrounding events	0.84 (0.47-1.51)	0.562
		Learning about nature, animals, or plants	2.10 (1.20-3.67)	0.010
	Peer problems in the abnormal range	Reading picture books	1.34 (0.54-3.31)	0.531
		Teaching number concepts	1.36 (0.80-2.32)	0.254
		Drawing or painting	0.69 (0.36-1.32)	0.259
		Cognitive games	1.17 (0.63-2.19)	0.618
		Learning characters in daily life	0.99 (0.61-1.61)	0.961
		Songs, poems, or nursery rhymes	1.67 (0.88-3.18)	0.116
		Storytelling	1.16 (0.70-1.93)	0.555

Emotional-behavioral or neurodevelopmental problem group	Specific emotional-behavioral problem or neurodevelopmental delay	Parent–child interactive activity	aOR (95% CI)	*p* value
		Handicrafts	0.87 (0.39-1.92)	0.729
		Physical activity	0.98 (0.58-1.65)	0.945
		Self-care skills	0.81 (0.40-1.64)	0.561
		Discussing surrounding events	0.93 (0.55-1.57)	0.777
		Learning about nature, animals, or plants	1.76 (1.02-3.04)	0.042
	Prosocial behavior difficulties in the abnormal range	Reading picture books	1.94 (0.88-4.29)	0.103
		Teaching number concepts	1.21 (0.71-2.07)	0.481
		Drawing or painting	0.86 (0.48-1.55)	0.625
		Cognitive games	1.46 (0.81-2.61)	0.205
		Learning characters in daily life	1.13 (0.70-1.82)	0.614
		Songs, poems, or nursery rhymes	0.78 (0.36-1.68)	0.525
		Storytelling	0.79 (0.45-1.40)	0.421
		Handicrafts	0.90 (0.41-1.97)	0.787
		Physical activity	0.90 (0.52-1.54)	0.689
		Self-care skills	1.02 (0.53-1.98)	0.953
		Discussing surrounding events	1.28 (0.79-2.06)	0.312
		Learning about nature, animals, or plants	0.95 (0.48-1.88)	0.877
Neurodevelopmental delay	Communication delay	Reading picture books	1.38 (0.39-4.84)	0.620
		Teaching number concepts	0.82 (0.32-2.11)	0.674
		Drawing or painting	0.89 (0.38-2.09)	0.789
		Cognitive games	1.25 (0.52-3.00)	0.620
		Learning characters in daily life	0.87 (0.41-1.84)	0.719
		Songs, poems, or nursery rhymes	1.15 (0.40-3.36)	0.793

Emotional-behavioral or neurodevelopmental problem group	Specific emotional-behavioral problem or neurodevelopmental delay	Parent–child interactive activity	aOR (95% CI)	*p* value
		Storytelling	1.02 (0.43-2.39)	0.966
		Handicrafts	NA	NA
		Physical activity	2.80 (1.35-5.82)	0.006
		Self-care skills	0.72 (0.22-2.32)	0.577
		Discussing surrounding events	0.67 (0.27-1.64)	0.381
		Learning about nature, animals, or plants	1.15 (0.47-2.84)	0.758
	Gross motor delay	Reading picture books	1.69 (0.49-5.89)	0.408
		Teaching number concepts	1.03 (0.40-2.62)	0.957
		Drawing or painting	0.67 (0.22-2.04)	0.477
		Cognitive games	1.18 (0.45-3.14)	0.737
		Learning characters in daily life	0.97 (0.44-2.14)	0.938
		Songs, poems, or nursery rhymes	2.47 (0.90-6.76)	0.078
		Storytelling	1.34 (0.59-3.07)	0.486
		Handicrafts	0.40 (0.06-2.93)	0.370
		Physical activity	1.16 (0.50-2.68)	0.733
		Self-care skills	1.10 (0.38-3.23)	0.858
		Discussing surrounding events	0.76 (0.30-1.93)	0.565
		Learning about nature, animals, or plants	0.37 (0.05-2.61)	0.320
	Fine motor delay	Reading picture books	1.33 (0.46-3.80)	0.599
		Teaching number concepts	1.06 (0.54-2.09)	0.857
		Drawing or painting	0.87 (0.44-1.74)	0.702
		Cognitive games	0.89 (0.38-2.04)	0.775
		Learning characters in daily life	0.66 (0.34-1.26)	0.207

**Emotional-behavioral or neurodevelopmental problem group**	**Specific emotional-behavioral problem or neurodevelopmental delay**	**Parent–child interactive activity**	**aOR (95% CI)**	***p* value**
		Songs, poems, or nursery rhymes	0.70 (0.28-1.80)	0.465
		Storytelling	0.73 (0.36-1.47)	0.377
		Handicrafts	0.76 (0.27-2.12)	0.602
		Physical activity	1.64 (0.92-2.92)	0.096
		Self-care skills	0.87 (0.38-2.01)	0.743
		Discussing surrounding events	0.94 (0.50-1.76)	0.840
		Learning about nature, animals, or plants	0.49 (0.15-1.57)	0.230
	Problem-solving delay	Reading picture books	1.15 (0.35-3.76)	0.821
		Teaching number concepts	0.74 (0.34-1.62)	0.456
		Drawing or painting	1.03 (0.52-2.03)	0.943
		Cognitive games	1.33 (0.60-2.96)	0.488
		Learning characters in daily life	0.96 (0.52-1.79)	0.902
		Songs, poems, or nursery rhymes	1.27 (0.51-3.14)	0.609
		Storytelling	0.54 (0.23-1.28)	0.161
		Handicrafts	0.78 (0.28-2.22)	0.647
		Physical activity	1.68 (0.92-3.07)	0.090
		Self-care skills	0.86 (0.35-2.11)	0.746
		Discussing surrounding events	1.26 (0.67-2.35)	0.469
		Learning about nature, animals, or plants	0.98 (0.42-2.32)	0.970
	Personal-social delay	Reading picture books	1.59 (0.47-5.41)	0.461
		Teaching number concepts	0.98 (0.40-2.40)	0.966
		Drawing or painting	0.86 (0.34-2.18)	0.744
		Cognitive games	1.69 (0.75-3.84)	0.206

Emotional-behavioral or neurodevelopmental problem group	Specific emotional-behavioral problem or neurodevelopmental delay	Parent–child interactive activity	aOR (95% CI)	*p* value
		Learning characters in daily life	0.65 (0.25-1.67)	0.370
		Songs, poems, or nursery rhymes	0.99 (0.32-3.09)	0.993
		Storytelling	0.43 (0.13-1.40)	0.160
		Handicrafts	1.94 (0.76-4.99)	0.167
		Physical activity	1.53 (0.73-3.22)	0.262
		Self-care skills	0.89 (0.28-2.80)	0.835
		Discussing surrounding events	0.80 (0.34-1.89)	0.607
		Learning about nature, animals, or plants	0.67 (0.18-2.55)	0.561

Note. Values are primary adjusted odds ratios per additional wave of low-level exposure to the specific activity. NA indicates that an estimate was not reported because of an unstable model estimate.

Supplementary Table S4. Complete fully adjusted estimates for overall and content-domain parent–child interaction frequency trajectories and cumulative low-level exposure in relation to abnormal total emotional and behavioral difficulties, specific emotional and behavioral problems, screening-positive delay in any neurodevelopmental domain, and specific neurodevelopmental delays.

Emotional-behavioral or neurodevelopmental problem group	Specific emotional-behavioral problem or neurodevelopmental delay	Parent–child interaction measure	Exposure form	Exposure contrast	aOR (95% CI)	*p* value
Emotional and behavioral problems	Abnormal total emotional and behavioral difficulties	Overall parent–child interaction frequency	Trajectory pattern	Deteriorated trajectory vs persistently normal	1.88 (0.57-6.15)	0.298
				Fluctuating trajectory vs persistently normal	1.67 (0.39-7.22)	0.491
				Improved trajectory vs persistently normal	1.04 (0.21-5.22)	0.966
				Persistently low trajectory vs persistently normal	3.27 (0.92-11.62)	0.067
			Cumulative low-level exposure	Per additional wave of low-level exposure	1.51 (0.99-2.31)	0.057
		Knowledge-learning interaction frequency	Trajectory pattern	Deteriorated trajectory vs persistently normal	1.11 (0.33-3.75)	0.872
				Fluctuating trajectory vs persistently normal	0.36 (0.07-1.77)	0.206
				Improved trajectory vs persistently normal	0.60 (0.18-1.96)	0.396
				Persistently low trajectory vs persistently normal	1.74 (0.42-7.23)	0.444
			Cumulative low-level exposure	Per additional wave of low-level exposure	1.10 (0.69-1.74)	0.688
		Story-reading interaction frequency	Trajectory pattern	Deteriorated trajectory vs persistently normal	0.92 (0.18-4.58)	0.916
				Fluctuating trajectory vs persistently normal	3.28 (1.09-9.87)	0.035
				Improved trajectory vs persistently normal	0.63 (0.17-2.37)	0.494

Emotional-behavioral or neurodevelopmental problem group	Specific emotional-behavioral problem or neurodevelopmental delay	Parent–child interaction measure	Exposure form	Exposure contrast	aOR (95% CI)	*p* value
				Persistently low trajectory vs persistently normal	6.24 (1.21-32.24)	0.029
			Cumulative low-level exposure	Per additional wave of low-level exposure	1.47 (0.92-2.36)	0.109
		Physical/cultural interaction frequency	Trajectory pattern	Deteriorated trajectory vs persistently normal	0.90 (0.32-2.53)	0.843
				Fluctuating trajectory vs persistently normal	1.09 (0.33-3.58)	0.887
				Improved trajectory vs persistently normal	0.41 (0.05-3.44)	0.413
				Persistently low trajectory vs persistently normal	2.27 (0.49-10.42)	0.293
			Cumulative low-level exposure	Per additional wave of low-level exposure	1.16 (0.73-1.83)	0.531
		Environmental interaction frequency	Trajectory pattern	Deteriorated trajectory vs persistently normal	1.62 (0.39-6.76)	0.507
				Fluctuating trajectory vs persistently normal	1.54 (0.43-5.49)	0.502
				Improved trajectory vs persistently normal	1.35 (0.40-4.52)	0.626
				Persistently low trajectory vs persistently normal	11.06 (1.90-64.49)	0.008
			Cumulative low-level exposure	Per additional wave of low-level exposure	1.54 (0.96-2.48)	0.075
Neurodevelopmental delay	Screening-positive delay in any neurodevelopmental domain	Overall parent–child interaction frequency	Trajectory pattern	Deteriorated trajectory vs persistently normal	0.53 (0.13-2.11)	0.368
				Fluctuating trajectory vs persistently normal	0.48 (0.09-2.59)	0.391

Emotional-behavioral or neurodevelopmental problem group	Specific emotional-behavioral problem or neurodevelopmental delay	Parent–child interaction measure	Exposure form	Exposure contrast	aOR (95% CI)	*p* value
				Improved trajectory vs persistently normal	1.31 (0.36-4.73)	0.684
				Persistently low trajectory vs persistently normal	0.52 (0.12-2.27)	0.385
			Cumulative low-level exposure	Per additional wave of low-level exposure	0.88 (0.55-1.39)	0.585
		Knowledge-learning interaction frequency	Trajectory pattern	Deteriorated trajectory vs persistently normal	0.71 (0.18-2.83)	0.628
				Fluctuating trajectory vs persistently normal	0.99 (0.30-3.27)	0.992
				Improved trajectory vs persistently normal	0.55 (0.17-1.82)	0.330
				Persistently low trajectory vs persistently normal	0.27 (0.03-2.54)	0.255
			Cumulative low-level exposure	Per additional wave of low-level exposure	0.65 (0.38-1.10)	0.109
		Story-reading interaction frequency	Trajectory pattern	Deteriorated trajectory vs persistently normal	1.35 (0.34-5.34)	0.669
				Fluctuating trajectory vs persistently normal	0.99 (0.25-4.02)	0.994
				Improved trajectory vs persistently normal	0.99 (0.32-3.02)	0.981
				Persistently low trajectory vs persistently normal	NA	NA
			Cumulative low-level exposure	Per additional wave of low-level exposure	0.83 (0.47-1.47)	0.529
		Physical/cultural interaction frequency	Trajectory pattern	Deteriorated trajectory vs persistently normal	1.02 (0.37-2.78)	0.972

Emotional-behavioral or neurodevelopmental problem group	Specific emotional-behavioral problem or neurodevelopmental delay	Parent–child interaction measure	Exposure form	Exposure contrast	aOR (95% CI)	*p* value
				Fluctuating trajectory vs persistently normal	0.35 (0.07-1.80)	0.210
				Improved trajectory vs persistently normal	0.43 (0.05-3.59)	0.435
				Persistently low trajectory vs persistently normal	1.77 (0.38-8.18)	0.467
			Cumulative low-level exposure	Per additional wave of low-level exposure	0.92 (0.57-1.51)	0.753
		Environmental interaction frequency	Trajectory pattern	Deteriorated trajectory vs persistently normal	0.36 (0.04-3.11)	0.353
				Fluctuating trajectory vs persistently normal	1.12 (0.35-3.62)	0.844
				Improved trajectory vs persistently normal	0.41 (0.08-1.99)	0.270
				Persistently low trajectory vs persistently normal	1.33 (0.13-13.27)	0.807
			Cumulative low-level exposure	Per additional wave of low-level exposure	0.72 (0.40-1.32)	0.290
Emotional and behavioral problems	Emotional symptoms in the abnormal range	Overall parent–child interaction frequency	Trajectory pattern	Deteriorated trajectory vs persistently normal	0.99 (0.27-3.67)	0.984
				Fluctuating trajectory vs persistently normal	0.79 (0.14-4.48)	0.794
				Improved trajectory vs persistently normal	0.97 (0.22-4.31)	0.967
				Persistently low trajectory vs persistently normal	0.66 (0.14-3.16)	0.600
			Cumulative low-level exposure	Per additional wave of low-level exposure	0.96 (0.59-1.57)	0.879

Emotional-behavioral or neurodevelopmental problem group	Specific emotional-behavioral problem or neurodevelopmental delay	Parent–child interaction measure	Exposure form	Exposure contrast	aOR (95% CI)	*p* value
		Knowledge-learning interaction frequency	Trajectory pattern	Deteriorated trajectory vs persistently normal	0.76 (0.18-3.16)	0.704
				Fluctuating trajectory vs persistently normal	0.14 (0.02-1.31)	0.086
				Improved trajectory vs persistently normal	0.58 (0.17-2.00)	0.391
				Persistently low trajectory vs persistently normal	0.90 (0.15-5.33)	0.906
			Cumulative low-level exposure	Per additional wave of low-level exposure	0.82 (0.47-1.42)	0.469
		Story-reading interaction frequency	Trajectory pattern	Deteriorated trajectory vs persistently normal	NA	NA
				Fluctuating trajectory vs persistently normal	2.12 (0.60-7.52)	0.242
				Improved trajectory vs persistently normal	0.28 (0.06-1.37)	0.116
				Persistently low trajectory vs persistently normal	1.17 (0.11-12.36)	0.897
			Cumulative low-level exposure	Per additional wave of low-level exposure	0.80 (0.43-1.50)	0.485
		Physical/cultural interaction frequency	Trajectory pattern	Deteriorated trajectory vs persistently normal	0.38 (0.10-1.43)	0.151
				Fluctuating trajectory vs persistently normal	0.64 (0.17-2.45)	0.518
				Improved trajectory vs persistently normal	0.35 (0.04-3.18)	0.351
				Persistently low trajectory vs persistently normal	1.33 (0.22-8.08)	0.753

Emotional-behavioral or neurodevelopmental problem group	Specific emotional-behavioral problem or neurodevelopmental delay	Parent–child interaction measure	Exposure form	Exposure contrast	aOR (95% CI)	*p* value
			Cumulative low-level exposure	Per additional wave of low-level exposure	0.90 (0.52-1.56)	0.708
		Environmental interaction frequency	Trajectory pattern	Deteriorated trajectory vs persistently normal	1.42 (0.30-6.82)	0.661
				Fluctuating trajectory vs persistently normal	0.73 (0.13-4.17)	0.724
				Improved trajectory vs persistently normal	1.67 (0.47-6.00)	0.429
				Persistently low trajectory vs persistently normal	7.45 (0.97-57.01)	0.053
			Cumulative low-level exposure	Per additional wave of low-level exposure	1.28 (0.74-2.24)	0.378
	Conduct problems in the abnormal range	Overall parent–child interaction frequency	Trajectory pattern	Deteriorated trajectory vs persistently normal	2.84 (0.88-9.20)	0.081
				Fluctuating trajectory vs persistently normal	1.90 (0.42-8.51)	0.401
				Improved trajectory vs persistently normal	1.99 (0.46-8.50)	0.354
				Persistently low trajectory vs persistently normal	3.66 (1.00-13.34)	0.049
			Cumulative low-level exposure	Per additional wave of low-level exposure	1.50 (0.98-2.30)	0.061
		Knowledge-learning interaction frequency	Trajectory pattern	Deteriorated trajectory vs persistently normal	0.74 (0.18-3.00)	0.676
				Fluctuating trajectory vs persistently normal	0.29 (0.06-1.54)	0.147
				Improved trajectory vs persistently normal	0.60 (0.18-2.01)	0.409

Emotional-behavioral or neurodevelopmental problem group	Specific emotional-behavioral problem or neurodevelopmental delay	Parent–child interaction measure	Exposure form	Exposure contrast	aOR (95% CI)	*p* value
				Persistently low trajectory vs persistently normal	2.34 (0.62-8.85)	0.209
			Cumulative low-level exposure	Per additional wave of low-level exposure	1.26 (0.80-1.97)	0.320
		Story-reading interaction frequency	Trajectory pattern	Deteriorated trajectory vs persistently normal	0.79 (0.15-4.16)	0.779
				Fluctuating trajectory vs persistently normal	1.76 (0.50-6.21)	0.379
				Improved trajectory vs persistently normal	1.15 (0.40-3.32)	0.801
				Persistently low trajectory vs persistently normal	5.11 (0.98-26.65)	0.053
			Cumulative low-level exposure	Per additional wave of low-level exposure	1.41 (0.88-2.26)	0.150
		Physical/cultural interaction frequency	Trajectory pattern	Deteriorated trajectory vs persistently normal	0.74 (0.26-2.16)	0.588
				Fluctuating trajectory vs persistently normal	0.53 (0.14-2.02)	0.349
				Improved trajectory vs persistently normal	0.67 (0.13-3.53)	0.640
				Persistently low trajectory vs persistently normal	1.59 (0.33-7.54)	0.561
			Cumulative low-level exposure	Per additional wave of low-level exposure	0.93 (0.57-1.50)	0.754
		Environmental interaction frequency	Trajectory pattern	Deteriorated trajectory vs persistently normal	0.70 (0.13-3.65)	0.673
				Fluctuating trajectory vs persistently normal	1.14 (0.32-4.12)	0.842

**Emotional-behavioral or neurodevelopmental problem group**	**Specific emotional-behavioral problem or neurodevelopmental delay**	**Parent–child interaction measure**	**Exposure form**	**Exposure contrast**	**aOR (95% CI)**	***p* value**
				Improved trajectory vs persistently normal	1.32 (0.42-4.13)	0.630
				Persistently low trajectory vs persistently normal	7.19 (1.24-41.75)	0.028
			Cumulative low-level exposure	Per additional wave of low-level exposure	1.45 (0.91-2.32)	0.117
	Hyperactivity/inattention in the abnormal range	Overall parent–child interaction frequency	Trajectory pattern	Deteriorated trajectory vs persistently normal	2.24 (0.58-8.62)	0.242
				Fluctuating trajectory vs persistently normal	1.47 (0.24-9.04)	0.680
				Improved trajectory vs persistently normal	2.79 (0.64-12.25)	0.174
				Persistently low trajectory vs persistently normal	3.20 (0.73-13.99)	0.122
			Cumulative low-level exposure	Per additional wave of low-level exposure	1.50 (0.92-2.44)	0.103
		Knowledge-learning interaction frequency	Trajectory pattern	Deteriorated trajectory vs persistently normal	1.13 (0.28-4.55)	0.867
				Fluctuating trajectory vs persistently normal	0.17 (0.02-1.60)	0.121
				Improved trajectory vs persistently normal	0.98 (0.31-3.12)	0.970
				Persistently low trajectory vs persistently normal	0.86 (0.15-5.13)	0.871
			Cumulative low-level exposure	Per additional wave of low-level exposure	1.00 (0.59-1.70)	0.998
		Story-reading interaction frequency	Trajectory pattern	Deteriorated trajectory vs persistently normal	0.64 (0.07-5.67)	0.685

Emotional-behavioral or neurodevelopmental problem group	Specific emotional-behavioral problem or neurodevelopmental delay	Parent–child interaction measure	Exposure form	Exposure contrast	aOR (95% CI)	*p* value
				Fluctuating trajectory vs persistently normal	2.85 (0.75-10.76)	0.123
				Improved trajectory vs persistently normal	1.35 (0.37-4.93)	0.649
				Persistently low trajectory vs persistently normal	9.26 (1.67-51.24)	0.011
			Cumulative low-level exposure	Per additional wave of low-level exposure	1.49 (0.88-2.54)	0.138
		Physical/cultural interaction frequency	Trajectory pattern	Deteriorated trajectory vs persistently normal	1.71 (0.54-5.43)	0.360
				Fluctuating trajectory vs persistently normal	1.83 (0.44-7.59)	0.408
				Improved trajectory vs persistently normal	NA	NA
				Persistently low trajectory vs persistently normal	8.63 (1.72-43.43)	0.009
			Cumulative low-level exposure	Per additional wave of low-level exposure	1.61 (0.96-2.71)	0.070
		Environmental interaction frequency	Trajectory pattern	Deteriorated trajectory vs persistently normal	1.88 (0.40-8.76)	0.422
				Fluctuating trajectory vs persistently normal	0.79 (0.14-4.49)	0.786
				Improved trajectory vs persistently normal	0.77 (0.16-3.86)	0.756
				Persistently low trajectory vs persistently normal	11.25 (1.79-70.90)	0.010
			Cumulative low-level exposure	Per additional wave of low-level exposure	1.62 (0.95-2.77)	0.075

Emotional-behavioral or neurodevelopmental problem group	Specific emotional-behavioral problem or neurodevelopmental delay	Parent–child interaction measure	Exposure form	Exposure contrast	aOR (95% CI)	*p* value
	Peer problems in the abnormal range	Overall parent–child interaction frequency	Trajectory pattern	Deteriorated trajectory vs persistently normal	1.07 (0.26-4.42)	0.930
				Fluctuating trajectory vs persistently normal	1.73 (0.38-7.85)	0.480
				Improved trajectory vs persistently normal	2.38 (0.64-8.89)	0.196
				Persistently low trajectory vs persistently normal	2.32 (0.58-9.25)	0.233
			Cumulative low-level exposure	Per additional wave of low-level exposure	1.44 (0.92-2.27)	0.111
		Knowledge-learning interaction frequency	Trajectory pattern	Deteriorated trajectory vs persistently normal	1.23 (0.34-4.39)	0.750
				Fluctuating trajectory vs persistently normal	0.19 (0.02-1.64)	0.131
				Improved trajectory vs persistently normal	0.75 (0.22-2.53)	0.644
				Persistently low trajectory vs persistently normal	2.27 (0.51-10.14)	0.285
			Cumulative low-level exposure	Per additional wave of low-level exposure	1.09 (0.67-1.79)	0.725
		Story-reading interaction frequency	Trajectory pattern	Deteriorated trajectory vs persistently normal	0.70 (0.13-3.69)	0.674
				Fluctuating trajectory vs persistently normal	1.81 (0.51-6.48)	0.361
				Improved trajectory vs persistently normal	0.68 (0.21-2.26)	0.531
				Persistently low trajectory vs persistently normal	NA	NA

Emotional-behavioral or neurodevelopmental problem group	Specific emotional-behavioral problem or neurodevelopmental delay	Parent–child interaction measure	Exposure form	Exposure contrast	aOR (95% CI)	*p* value
			Cumulative low-level exposure	Per additional wave of low-level exposure	0.83 (0.46-1.51)	0.551
		Physical/cultural interaction frequency	Trajectory pattern	Deteriorated trajectory vs persistently normal	0.43 (0.11-1.62)	0.212
				Fluctuating trajectory vs persistently normal	1.33 (0.44-4.01)	0.608
				Improved trajectory vs persistently normal	0.49 (0.06-4.13)	0.515
				Persistently low trajectory vs persistently normal	1.10 (0.20-6.20)	0.913
			Cumulative low-level exposure	Per additional wave of low-level exposure	0.94 (0.58-1.55)	0.823
		Environmental interaction frequency	Trajectory pattern	Deteriorated trajectory vs persistently normal	1.22 (0.23-6.56)	0.820
				Fluctuating trajectory vs persistently normal	2.11 (0.57-7.78)	0.262
				Improved trajectory vs persistently normal	2.10 (0.65-6.79)	0.216
				Persistently low trajectory vs persistently normal	8.86 (1.18-66.49)	0.034
			Cumulative low-level exposure	Per additional wave of low-level exposure	1.50 (0.90-2.50)	0.121
	Prosocial behavior difficulties in the abnormal range	Overall parent–child interaction frequency	Trajectory pattern	Deteriorated trajectory vs persistently normal	1.78 (0.49-6.44)	0.376
				Fluctuating trajectory vs persistently normal	2.00 (0.45-8.86)	0.364
				Improved trajectory vs persistently normal	1.30 (0.26-6.56)	0.751

Emotional-behavioral or neurodevelopmental problem group	Specific emotional-behavioral problem or neurodevelopmental delay	Parent–child interaction measure	Exposure form	Exposure contrast	aOR (95% CI)	*p* value
				Persistently low trajectory vs persistently normal	3.54 (0.94-13.26)	0.061
			Cumulative low-level exposure	Per additional wave of low-level exposure	1.50 (0.97-2.34)	0.071
		Knowledge-learning interaction frequency	Trajectory pattern	Deteriorated trajectory vs persistently normal	0.64 (0.13-3.12)	0.581
				Fluctuating trajectory vs persistently normal	0.24 (0.03-2.04)	0.193
				Improved trajectory vs persistently normal	1.05 (0.35-3.17)	0.937
				Persistently low trajectory vs persistently normal	3.17 (0.79-12.75)	0.103
			Cumulative low-level exposure	Per additional wave of low-level exposure	1.51 (0.95-2.39)	0.083
		Story-reading interaction frequency	Trajectory pattern	Deteriorated trajectory vs persistently normal	0.98 (0.20-4.89)	0.983
				Fluctuating trajectory vs persistently normal	0.82 (0.17-3.99)	0.801
				Improved trajectory vs persistently normal	1.70 (0.62-4.65)	0.298
				Persistently low trajectory vs persistently normal	NA	NA
			Cumulative low-level exposure	Per additional wave of low-level exposure	1.17 (0.70-1.97)	0.543
		Physical/cultural interaction frequency	Trajectory pattern	Deteriorated trajectory vs persistently normal	0.86 (0.30-2.45)	0.783
				Fluctuating trajectory vs persistently normal	0.54 (0.13-2.19)	0.385

Emotional-behavioral or neurodevelopmental problem group	Specific emotional-behavioral problem or neurodevelopmental delay	Parent–child interaction measure	Exposure form	Exposure contrast	aOR (95% CI)	*p* value
				Improved trajectory vs persistently normal	0.41 (0.05-3.47)	0.416
				Persistently low trajectory vs persistently normal	1.07 (0.19-5.97)	0.938
			Cumulative low-level exposure	Per additional wave of low-level exposure	0.92 (0.56-1.51)	0.740
		Environmental interaction frequency	Trajectory pattern	Deteriorated trajectory vs persistently normal	1.16 (0.28-4.80)	0.837
				Fluctuating trajectory vs persistently normal	0.50 (0.10-2.48)	0.399
				Improved trajectory vs persistently normal	0.70 (0.19-2.62)	0.593
				Persistently low trajectory vs persistently normal	NA	NA
			Cumulative low-level exposure	Per additional wave of low-level exposure	0.81 (0.45-1.46)	0.485
Neurodevelopmental delay	Communication delay	Overall parent–child interaction frequency	Trajectory pattern	Deteriorated trajectory vs persistently normal	NA	NA
				Fluctuating trajectory vs persistently normal	0.97 (0.08-12.14)	0.981
				Improved trajectory vs persistently normal	1.33 (0.12-14.21)	0.815
				Persistently low trajectory vs persistently normal	1.64 (0.19-14.30)	0.655
			Cumulative low-level exposure	Per additional wave of low-level exposure	1.18 (0.57-2.45)	0.664
		Knowledge-learning interaction frequency	Trajectory pattern	Deteriorated trajectory vs persistently normal	0.89 (0.08-9.62)	0.920

Emotional-behavioral or neurodevelopmental problem group	Specific emotional-behavioral problem or neurodevelopmental delay	Parent–child interaction measure	Exposure form	Exposure contrast	aOR (95% CI)	*p* value
				Fluctuating trajectory vs persistently normal	2.13 (0.35-13.02)	0.414
				Improved trajectory vs persistently normal	1.02 (0.17-6.03)	0.980
				Persistently low trajectory vs persistently normal	0.71 (0.05-10.13)	0.799
			Cumulative low-level exposure	Per additional wave of low-level exposure	0.87 (0.41-1.85)	0.718
		Story-reading interaction frequency	Trajectory pattern	Deteriorated trajectory vs persistently normal	0.81 (0.08-8.73)	0.862
				Fluctuating trajectory vs persistently normal	0.52 (0.04-6.78)	0.620
				Improved trajectory vs persistently normal	1.26 (0.25-6.36)	0.783
				Persistently low trajectory vs persistently normal	NA	NA
			Cumulative low-level exposure	Per additional wave of low-level exposure	0.79 (0.33-1.88)	0.601
		Physical/cultural interaction frequency	Trajectory pattern	Deteriorated trajectory vs persistently normal	0.70 (0.11-4.35)	0.704
				Fluctuating trajectory vs persistently normal	0.23 (0.02-2.97)	0.260
				Improved trajectory vs persistently normal	1.28 (0.11-14.28)	0.842
				Persistently low trajectory vs persistently normal	1.30 (0.10-16.47)	0.838
			Cumulative low-level exposure	Per additional wave of low-level exposure	0.83 (0.37-1.89)	0.659

**Emotional-behavioral or neurodevelopmental problem group**	**Specific emotional-behavioral problem or neurodevelopmental delay**	**Parent–child interaction measure**	**Exposure form**	**Exposure contrast**	**aOR (95% CI)**	***p* value**
		Environmental interaction frequency	Trajectory pattern	Deteriorated trajectory vs persistently normal	0.91 (0.08-10.20)	0.938
				Fluctuating trajectory vs persistently normal	0.84 (0.14-5.19)	0.854
				Improved trajectory vs persistently normal	0.52 (0.05-4.92)	0.566
				Persistently low trajectory vs persistently normal	NA	NA
			Cumulative low-level exposure	Per additional wave of low-level exposure	0.64 (0.25-1.66)	0.359
	Gross motor delay	Overall parent–child interaction frequency	Trajectory pattern	Deteriorated trajectory vs persistently normal	0.61 (0.07-5.15)	0.648
				Fluctuating trajectory vs persistently normal	NA	NA
				Improved trajectory vs persistently normal	1.35 (0.16-11.72)	0.783
				Persistently low trajectory vs persistently normal	0.36 (0.03-4.80)	0.437
			Cumulative low-level exposure	Per additional wave of low-level exposure	1.10 (0.52-2.30)	0.810
		Knowledge-learning interaction frequency	Trajectory pattern	Deteriorated trajectory vs persistently normal	0.49 (0.04-5.86)	0.571
				Fluctuating trajectory vs persistently normal	0.55 (0.05-6.15)	0.626
				Improved trajectory vs persistently normal	0.27 (0.03-2.96)	0.286
				Persistently low trajectory vs persistently normal	1.13 (0.09-14.95)	0.927

Emotional-behavioral or neurodevelopmental problem group	Specific emotional-behavioral problem or neurodevelopmental delay	Parent–child interaction measure	Exposure form	Exposure contrast	aOR (95% CI)	*p* value
			Cumulative low-level exposure	Per additional wave of low-level exposure	0.79 (0.34-1.84)	0.588
		Story-reading interaction frequency	Trajectory pattern	Deteriorated trajectory vs persistently normal	1.27 (0.10-16.79)	0.856
				Fluctuating trajectory vs persistently normal	3.00 (0.50-18.20)	0.231
				Improved trajectory vs persistently normal	NA	NA
				Persistently low trajectory vs persistently normal	NA	NA
			Cumulative low-level exposure	Per additional wave of low-level exposure	0.84 (0.35-2.02)	0.692
		Physical/cultural interaction frequency	Trajectory pattern	Deteriorated trajectory vs persistently normal	1.62 (0.31-8.64)	0.569
				Fluctuating trajectory vs persistently normal	0.57 (0.05-6.80)	0.654
				Improved trajectory vs persistently normal	NA	NA
				Persistently low trajectory vs persistently normal	0.77 (0.06-10.14)	0.841
			Cumulative low-level exposure	Per additional wave of low-level exposure	0.93 (0.42-2.04)	0.848
		Environmental interaction frequency	Trajectory pattern	Deteriorated trajectory vs persistently normal	NA	NA
				Fluctuating trajectory vs persistently normal	2.54 (0.32-20.41)	0.382
				Improved trajectory vs persistently normal	1.23 (0.11-13.97)	0.865

Emotional-behavioral or neurodevelopmental problem group	Specific emotional-behavioral problem or neurodevelopmental delay	Parent–child interaction measure	Exposure form	Exposure contrast	aOR (95% CI)	*p* value
				Persistently low trajectory vs persistently normal	NA	NA
			Cumulative low-level exposure	Per additional wave of low-level exposure	0.65 (0.22-1.88)	0.426
	Fine motor delay	Overall parent–child interaction frequency	Trajectory pattern	Deteriorated trajectory vs persistently normal	1.00 (0.23-4.37)	0.998
				Fluctuating trajectory vs persistently normal	0.75 (0.12-4.94)	0.769
				Improved trajectory vs persistently normal	0.98 (0.17-5.52)	0.979
				Persistently low trajectory vs persistently normal	0.38 (0.05-2.70)	0.334
			Cumulative low-level exposure	Per additional wave of low-level exposure	0.92 (0.52-1.62)	0.763
		Knowledge-learning interaction frequency	Trajectory pattern	Deteriorated trajectory vs persistently normal	0.93 (0.18-4.94)	0.932
				Fluctuating trajectory vs persistently normal	1.68 (0.44-6.42)	0.448
				Improved trajectory vs persistently normal	0.48 (0.10-2.40)	0.372
				Persistently low trajectory vs persistently normal	NA	NA
			Cumulative low-level exposure	Per additional wave of low-level exposure	0.57 (0.29-1.12)	0.104
		Story-reading interaction frequency	Trajectory pattern	Deteriorated trajectory vs persistently normal	0.99 (0.18-5.45)	0.988
				Fluctuating trajectory vs persistently normal	1.20 (0.25-5.73)	0.818

Emotional-behavioral or neurodevelopmental problem group	Specific emotional-behavioral problem or neurodevelopmental delay	Parent–child interaction measure	Exposure form	Exposure contrast	aOR (95% CI)	*p* value
				Improved trajectory vs persistently normal	0.20 (0.02-1.69)	0.139
				Persistently low trajectory vs persistently normal	NA	NA
			Cumulative low-level exposure	Per additional wave of low-level exposure	0.69 (0.33-1.44)	0.323
		Physical/cultural interaction frequency	Trajectory pattern	Deteriorated trajectory vs persistently normal	1.44 (0.45-4.56)	0.540
				Fluctuating trajectory vs persistently normal	0.45 (0.07-2.67)	0.377
				Improved trajectory vs persistently normal	0.77 (0.09-6.90)	0.815
				Persistently low trajectory vs persistently normal	NA	NA
			Cumulative low-level exposure	Per additional wave of low-level exposure	0.68 (0.35-1.33)	0.260
		Environmental interaction frequency	Trajectory pattern	Deteriorated trajectory vs persistently normal	0.66 (0.07-6.21)	0.715
				Fluctuating trajectory vs persistently normal	1.57 (0.41-5.98)	0.507
				Improved trajectory vs persistently normal	0.68 (0.13-3.61)	0.653
				Persistently low trajectory vs persistently normal	NA	NA
			Cumulative low-level exposure	Per additional wave of low-level exposure	0.63 (0.29-1.36)	0.237
	Problem-solving delay	Overall parent–child interaction frequency	Trajectory pattern	Deteriorated trajectory vs persistently normal	NA	NA

Emotional-behavioral or neurodevelopmental problem group	Specific emotional-behavioral problem or neurodevelopmental delay	Parent–child interaction measure	Exposure form	Exposure contrast	aOR (95% CI)	*p* value
				Fluctuating trajectory vs persistently normal	0.42 (0.04-4.24)	0.462
				Improved trajectory vs persistently normal	1.25 (0.21-7.31)	0.808
				Persistently low trajectory vs persistently normal	1.08 (0.19-6.03)	0.932
			Cumulative low-level exposure	Per additional wave of low-level exposure	0.96 (0.53-1.76)	0.907
		Knowledge-learning interaction frequency	Trajectory pattern	Deteriorated trajectory vs persistently normal	0.34 (0.04-3.19)	0.342
				Fluctuating trajectory vs persistently normal	0.71 (0.13-3.90)	0.697
				Improved trajectory vs persistently normal	0.87 (0.20-3.76)	0.854
				Persistently low trajectory vs persistently normal	0.51 (0.05-5.70)	0.585
			Cumulative low-level exposure	Per additional wave of low-level exposure	0.85 (0.44-1.66)	0.639
		Story-reading interaction frequency	Trajectory pattern	Deteriorated trajectory vs persistently normal	1.48 (0.27-8.26)	0.653
				Fluctuating trajectory vs persistently normal	0.74 (0.09-6.40)	0.782
				Improved trajectory vs persistently normal	1.01 (0.24-4.28)	0.991
				Persistently low trajectory vs persistently normal	NA	NA
			Cumulative low-level exposure	Per additional wave of low-level exposure	0.79 (0.36-1.73)	0.553

Emotional-behavioral or neurodevelopmental problem group	Specific emotional-behavioral problem or neurodevelopmental delay	Parent–child interaction measure	Exposure form	Exposure contrast	aOR (95% CI)	*p* value
		Physical/cultural interaction frequency	Trajectory pattern	Deteriorated trajectory vs persistently normal	0.78 (0.19-3.24)	0.731
				Fluctuating trajectory vs persistently normal	0.40 (0.04-3.76)	0.424
				Improved trajectory vs persistently normal	0.69 (0.07-6.40)	0.745
				Persistently low trajectory vs persistently normal	3.26 (0.48-22.05)	0.225
			Cumulative low-level exposure	Per additional wave of low-level exposure	1.03 (0.55-1.94)	0.930
		Environmental interaction frequency	Trajectory pattern	Deteriorated trajectory vs persistently normal	0.62 (0.06-6.19)	0.681
				Fluctuating trajectory vs persistently normal	1.46 (0.31-6.86)	0.634
				Improved trajectory vs persistently normal	0.52 (0.06-4.44)	0.548
				Persistently low trajectory vs persistently normal	2.78 (0.24-32.90)	0.417
			Cumulative low-level exposure	Per additional wave of low-level exposure	0.99 (0.48-2.04)	0.985
	Personal-social delay	Overall parent–child interaction frequency	Trajectory pattern	Deteriorated trajectory vs persistently normal	0.52 (0.05-5.00)	0.572
				Fluctuating trajectory vs persistently normal	NA	NA
				Improved trajectory vs persistently normal	1.98 (0.34-11.61)	0.448
				Persistently low trajectory vs persistently normal	0.42 (0.03-5.40)	0.506

Emotional-behavioral or neurodevelopmental problem group	Specific emotional-behavioral problem or neurodevelopmental delay	Parent–child interaction measure	Exposure form	Exposure contrast	aOR (95% CI)	*p* value
			Cumulative low-level exposure	Per additional wave of low-level exposure	0.78 (0.36-1.69)	0.525
		Knowledge-learning interaction frequency	Trajectory pattern	Deteriorated trajectory vs persistently normal	0.80 (0.08-7.47)	0.841
				Fluctuating trajectory vs persistently normal	1.29 (0.22-7.71)	0.779
				Improved trajectory vs persistently normal	0.43 (0.05-3.82)	0.451
				Persistently low trajectory vs persistently normal	NA	NA
			Cumulative low-level exposure	Per additional wave of low-level exposure	0.63 (0.25-1.57)	0.321
		Story-reading interaction frequency	Trajectory pattern	Deteriorated trajectory vs persistently normal	3.54 (0.59-21.06)	0.166
				Fluctuating trajectory vs persistently normal	1.27 (0.13-12.70)	0.841
				Improved trajectory vs persistently normal	1.48 (0.26-8.34)	0.658
				Persistently low trajectory vs persistently normal	NA	NA
			Cumulative low-level exposure	Per additional wave of low-level exposure	1.33 (0.60-2.98)	0.483
		Physical/cultural interaction frequency	Trajectory pattern	Deteriorated trajectory vs persistently normal	2.34 (0.49-11.04)	0.285
				Fluctuating trajectory vs persistently normal	1.19 (0.11-12.71)	0.886
				Improved trajectory vs persistently normal	NA	NA

Emotional-behavioral or neurodevelopmental problem group	Specific emotional-behavioral problem or neurodevelopmental delay	Parent–child interaction measure	Exposure form	Exposure contrast	aOR (95% CI)	*p* value
				Persistently low trajectory vs persistently normal	9.53 (1.06-85.66)	0.044
			Cumulative low-level exposure	Per additional wave of low-level exposure	1.55 (0.74-3.24)	0.241
		Environmental interaction frequency	Trajectory pattern	Deteriorated trajectory vs persistently normal	NA	NA
				Fluctuating trajectory vs persistently normal	NA	NA
				Improved trajectory vs persistently normal	0.64 (0.07-5.54)	0.684
				Persistently low trajectory vs persistently normal	3.72 (0.29-48.17)	0.315
			Cumulative low-level exposure	Per additional wave of low-level exposure	0.77 (0.29-2.05)	0.607

Note. Values are adjusted odds ratios from the fully adjusted logistic regression model. NA indicates that an estimate was not reported because of an unstable model estimate.

Supplementary Table S5. Complete Firth penalized estimates for overall and content-domain parent–child interaction frequency trajectories and cumulative low-level exposure in relation to abnormal total emotional and behavioral difficulties, specific emotional and behavioral problems, screening-positive delay in any neurodevelopmental domain, and specific neurodevelopmental delays.

Emotional-behavioral or neurodevelopmental problem group	Specific emotional-behavioral problem or neurodevelopmental delay	Parent–child interaction measure	Exposure form	Exposure contrast	aOR (95% CI)	*p* value
Emotional and behavioral problems	Abnormal total emotional and behavioral difficulties	Overall parent–child interaction frequency	Trajectory pattern	Deteriorated trajectory vs persistently normal	2.08 (0.63-6.06)	0.216
				Fluctuating trajectory vs persistently normal	1.87 (0.42-6.69)	0.385
				Improved trajectory vs persistently normal	1.28 (0.23-4.79)	0.741
				Persistently low trajectory vs persistently normal	3.37 (1.00-11.07)	0.050
			Cumulative low-level exposure	Per additional wave of low-level exposure	1.52 (1.01-2.28)	0.044
		Knowledge-learning interaction frequency	Trajectory pattern	Deteriorated trajectory vs persistently normal	1.24 (0.35-3.60)	0.722
				Fluctuating trajectory vs persistently normal	0.47 (0.09-1.72)	0.276
				Improved trajectory vs persistently normal	0.72 (0.21-1.99)	0.550
				Persistently low trajectory vs persistently normal	1.93 (0.48-6.83)	0.334
			Cumulative low-level exposure	Per additional wave of low-level exposure	1.14 (0.73-1.74)	0.568
		Story-reading interaction frequency	Trajectory pattern	Deteriorated trajectory vs persistently normal	1.14 (0.21-4.19)	0.864
				Fluctuating trajectory vs persistently normal	3.11 (1.03-8.58)	0.045
				Improved trajectory vs persistently normal	0.72 (0.18-2.22)	0.594

**Emotional-behavioral or neurodevelopmental problem group**	**Specific emotional-behavioral problem or neurodevelopmental delay**	**Parent–child interaction measure**	**Exposure form**	**Exposure contrast**	**aOR (95% CI)**	***p* value**
				Persistently low trajectory vs persistently normal	5.66 (1.17-24.45)	0.032
			Cumulative low-level exposure	Per additional wave of low-level exposure	1.44 (0.90-2.23)	0.121
		Physical/cultural interaction frequency	Trajectory pattern	Deteriorated trajectory vs persistently normal	0.97 (0.34-2.48)	0.953
				Fluctuating trajectory vs persistently normal	1.11 (0.34-3.26)	0.851
				Improved trajectory vs persistently normal	0.63 (0.07-2.80)	0.587
				Persistently low trajectory vs persistently normal	2.40 (0.53-9.02)	0.238
			Cumulative low-level exposure	Per additional wave of low-level exposure	1.17 (0.75-1.79)	0.482
		Environmental interaction frequency	Trajectory pattern	Deteriorated trajectory vs persistently normal	1.76 (0.41-6.04)	0.415
				Fluctuating trajectory vs persistently normal	1.64 (0.45-5.07)	0.426
				Improved trajectory vs persistently normal	1.42 (0.41-4.05)	0.553
				Persistently low trajectory vs persistently normal	9.78 (1.84-49.98)	0.009
			Cumulative low-level exposure	Per additional wave of low-level exposure	1.53 (0.95-2.41)	0.076
Neurodevelopmental delay	Screening-positive delay in any neurodevelopmental domain	Overall parent–child interaction frequency	Trajectory pattern	Deteriorated trajectory vs persistently normal	0.67 (0.16-2.19)	0.527
				Fluctuating trajectory vs persistently normal	0.63 (0.11-2.50)	0.531

**Emotional-behavioral or neurodevelopmental problem group**	**Specific emotional-behavioral problem or neurodevelopmental delay**	**Parent–child interaction measure**	**Exposure form**	**Exposure contrast**	**aOR (95% CI)**	***p* value**
				Improved trajectory vs persistently normal	1.52 (0.41-4.73)	0.501
				Persistently low trajectory vs persistently normal	0.60 (0.14-2.25)	0.459
			Cumulative low-level exposure	Per additional wave of low-level exposure	0.90 (0.58-1.38)	0.643
		Knowledge-learning interaction frequency	Trajectory pattern	Deteriorated trajectory vs persistently normal	0.92 (0.22-2.94)	0.891
				Fluctuating trajectory vs persistently normal	1.09 (0.33-3.15)	0.875
				Improved trajectory vs persistently normal	0.70 (0.20-1.94)	0.512
				Persistently low trajectory vs persistently normal	0.47 (0.05-2.34)	0.392
			Cumulative low-level exposure	Per additional wave of low-level exposure	0.72 (0.42-1.15)	0.177
		Story-reading interaction frequency	Trajectory pattern	Deteriorated trajectory vs persistently normal	1.34 (0.31-4.44)	0.666
				Fluctuating trajectory vs persistently normal	1.29 (0.31-4.10)	0.701
				Improved trajectory vs persistently normal	0.99 (0.31-2.70)	0.980
				Persistently low trajectory vs persistently normal	0.40 (0.00-3.67)	0.494
			Cumulative low-level exposure	Per additional wave of low-level exposure	0.87 (0.48-1.44)	0.594
		Physical/cultural interaction frequency	Trajectory pattern	Deteriorated trajectory vs persistently normal	1.16 (0.42-2.92)	0.759

Emotional-behavioral or neurodevelopmental problem group	Specific emotional-behavioral problem or neurodevelopmental delay	Parent–child interaction measure	Exposure form	Exposure contrast	aOR (95% CI)	*p* value
				Fluctuating trajectory vs persistently normal	0.45 (0.08-1.70)	0.258
				Improved trajectory vs persistently normal	0.62 (0.06-2.78)	0.570
				Persistently low trajectory vs persistently normal	2.48 (0.54-9.50)	0.226
			Cumulative low-level exposure	Per additional wave of low-level exposure	1.01 (0.62-1.59)	0.964
		Environmental interaction frequency	Trajectory pattern	Deteriorated trajectory vs persistently normal	0.47 (0.05-2.17)	0.370
				Fluctuating trajectory vs persistently normal	1.22 (0.38-3.47)	0.724
				Improved trajectory vs persistently normal	0.56 (0.11-1.88)	0.376
				Persistently low trajectory vs persistently normal	1.70 (0.16-9.74)	0.606
			Cumulative low-level exposure	Per additional wave of low-level exposure	0.77 (0.42-1.32)	0.363
Emotional and behavioral problems	Emotional symptoms in the abnormal range	Overall parent–child interaction frequency	Trajectory pattern	Deteriorated trajectory vs persistently normal	1.12 (0.31-3.47)	0.853
				Fluctuating trajectory vs persistently normal	0.85 (0.14-3.58)	0.838
				Improved trajectory vs persistently normal	1.22 (0.27-4.27)	0.778
				Persistently low trajectory vs persistently normal	0.76 (0.17-2.98)	0.697
			Cumulative low-level exposure	Per additional wave of low-level exposure	0.98 (0.61-1.53)	0.919

**Emotional-behavioral or neurodevelopmental problem group**	**Specific emotional-behavioral problem or neurodevelopmental delay**	**Parent–child interaction measure**	**Exposure form**	**Exposure contrast**	**aOR (95% CI)**	***p* value**
		Knowledge-learning interaction frequency	Trajectory pattern	Deteriorated trajectory vs persistently normal	0.85 (0.20-2.78)	0.806
				Fluctuating trajectory vs persistently normal	0.21 (0.02-1.05)	0.058
				Improved trajectory vs persistently normal	0.65 (0.19-1.86)	0.441
				Persistently low trajectory vs persistently normal	0.88 (0.15-3.84)	0.878
			Cumulative low-level exposure	Per additional wave of low-level exposure	0.82 (0.48-1.34)	0.436
		Story-reading interaction frequency	Trajectory pattern	Deteriorated trajectory vs persistently normal	0.15 (0.00-1.29)	0.095
				Fluctuating trajectory vs persistently normal	1.91 (0.55-5.82)	0.292
				Improved trajectory vs persistently normal	0.39 (0.07-1.36)	0.151
				Persistently low trajectory vs persistently normal	1.43 (0.14-7.83)	0.724
			Cumulative low-level exposure	Per additional wave of low-level exposure	0.82 (0.43-1.41)	0.484
		Physical/cultural interaction frequency	Trajectory pattern	Deteriorated trajectory vs persistently normal	0.42 (0.10-1.32)	0.146
				Fluctuating trajectory vs persistently normal	0.66 (0.17-2.13)	0.507
				Improved trajectory vs persistently normal	0.53 (0.05-2.47)	0.456
				Persistently low trajectory vs persistently normal	1.43 (0.23-6.35)	0.669

Emotional-behavioral or neurodevelopmental problem group	Specific emotional-behavioral problem or neurodevelopmental delay	Parent–child interaction measure	Exposure form	Exposure contrast	aOR (95% CI)	*p* value
			Cumulative low-level exposure	Per additional wave of low-level exposure	0.89 (0.51-1.45)	0.642
		Environmental interaction frequency	Trajectory pattern	Deteriorated trajectory vs persistently normal	1.51 (0.32-5.60)	0.576
				Fluctuating trajectory vs persistently normal	0.87 (0.16-3.40)	0.859
				Improved trajectory vs persistently normal	1.50 (0.42-4.50)	0.504
				Persistently low trajectory vs persistently normal	6.38 (0.91-35.95)	0.061
			Cumulative low-level exposure	Per additional wave of low-level exposure	1.26 (0.73-2.10)	0.392
	Conduct problems in the abnormal range	Overall parent–child interaction frequency	Trajectory pattern	Deteriorated trajectory vs persistently normal	2.95 (0.93-8.73)	0.066
				Fluctuating trajectory vs persistently normal	2.12 (0.46-7.88)	0.309
				Improved trajectory vs persistently normal	2.18 (0.49-7.74)	0.279
				Persistently low trajectory vs persistently normal	3.79 (1.12-12.76)	0.032
			Cumulative low-level exposure	Per additional wave of low-level exposure	1.51 (1.01-2.26)	0.042
		Knowledge-learning interaction frequency	Trajectory pattern	Deteriorated trajectory vs persistently normal	0.93 (0.22-3.03)	0.908
				Fluctuating trajectory vs persistently normal	0.41 (0.07-1.55)	0.204
				Improved trajectory vs persistently normal	0.72 (0.21-2.04)	0.552

Emotional-behavioral or neurodevelopmental problem group	Specific emotional-behavioral problem or neurodevelopmental delay	Parent–child interaction measure	Exposure form	Exposure contrast	aOR (95% CI)	*p* value
				Persistently low trajectory vs persistently normal	2.66 (0.76-8.94)	0.124
			Cumulative low-level exposure	Per additional wave of low-level exposure	1.30 (0.85-1.97)	0.227
		Story-reading interaction frequency	Trajectory pattern	Deteriorated trajectory vs persistently normal	0.98 (0.17-3.73)	0.977
				Fluctuating trajectory vs persistently normal	1.73 (0.47-5.34)	0.385
				Improved trajectory vs persistently normal	1.20 (0.41-3.19)	0.724
				Persistently low trajectory vs persistently normal	5.19 (1.06-22.47)	0.042
			Cumulative low-level exposure	Per additional wave of low-level exposure	1.39 (0.88-2.16)	0.154
		Physical/cultural interaction frequency	Trajectory pattern	Deteriorated trajectory vs persistently normal	0.85 (0.29-2.23)	0.745
				Fluctuating trajectory vs persistently normal	0.70 (0.18-2.24)	0.561
				Improved trajectory vs persistently normal	0.84 (0.15-3.17)	0.811
				Persistently low trajectory vs persistently normal	2.01 (0.42-7.88)	0.354
			Cumulative low-level exposure	Per additional wave of low-level exposure	1.00 (0.62-1.55)	0.993
		Environmental interaction frequency	Trajectory pattern	Deteriorated trajectory vs persistently normal	0.79 (0.14-3.04)	0.751
				Fluctuating trajectory vs persistently normal	1.16 (0.31-3.63)	0.807

**Emotional-behavioral or neurodevelopmental problem group**	**Specific emotional-behavioral problem or neurodevelopmental delay**	**Parent–child interaction measure**	**Exposure form**	**Exposure contrast**	**aOR (95% CI)**	***p* value**
				Improved trajectory vs persistently normal	1.45 (0.46-3.96)	0.499
				Persistently low trajectory vs persistently normal	6.39 (1.20-31.57)	0.031
			Cumulative low-level exposure	Per additional wave of low-level exposure	1.45 (0.91-2.26)	0.114
	Hyperactivity/inattention in the abnormal range	Overall parent–child interaction frequency	Trajectory pattern	Deteriorated trajectory vs persistently normal	2.25 (0.59-7.54)	0.222
				Fluctuating trajectory vs persistently normal	1.84 (0.30-8.21)	0.476
				Improved trajectory vs persistently normal	2.81 (0.63-10.28)	0.164
				Persistently low trajectory vs persistently normal	3.20 (0.78-12.70)	0.105
			Cumulative low-level exposure	Per additional wave of low-level exposure	1.48 (0.93-2.35)	0.096
		Knowledge-learning interaction frequency	Trajectory pattern	Deteriorated trajectory vs persistently normal	1.17 (0.27-3.88)	0.818
				Fluctuating trajectory vs persistently normal	0.32 (0.03-1.55)	0.172
				Improved trajectory vs persistently normal	1.19 (0.37-3.32)	0.750
				Persistently low trajectory vs persistently normal	1.17 (0.20-5.14)	0.844
			Cumulative low-level exposure	Per additional wave of low-level exposure	1.07 (0.63-1.73)	0.802
		Story-reading interaction frequency	Trajectory pattern	Deteriorated trajectory vs persistently normal	0.92 (0.09-4.49)	0.931

**Emotional-behavioral or neurodevelopmental problem group**	**Specific emotional-behavioral problem or neurodevelopmental delay**	**Parent–child interaction measure**	**Exposure form**	**Exposure contrast**	**aOR (95% CI)**	***p* value**
				Fluctuating trajectory vs persistently normal	2.84 (0.75-9.20)	0.118
				Improved trajectory vs persistently normal	1.43 (0.39-4.43)	0.564
				Persistently low trajectory vs persistently normal	8.76 (1.75-40.21)	0.010
			Cumulative low-level exposure	Per additional wave of low-level exposure	1.53 (0.91-2.48)	0.105
		Physical/cultural interaction frequency	Trajectory pattern	Deteriorated trajectory vs persistently normal	1.74 (0.56-4.94)	0.323
				Fluctuating trajectory vs persistently normal	1.51 (0.38-5.20)	0.539
				Improved trajectory vs persistently normal	0.35 (0.00-3.04)	0.415
				Persistently low trajectory vs persistently normal	5.97 (1.37-24.19)	0.019
			Cumulative low-level exposure	Per additional wave of low-level exposure	1.52 (0.93-2.43)	0.090
		Environmental interaction frequency	Trajectory pattern	Deteriorated trajectory vs persistently normal	1.91 (0.41-7.18)	0.384
				Fluctuating trajectory vs persistently normal	1.06 (0.19-4.20)	0.941
				Improved trajectory vs persistently normal	0.96 (0.18-3.53)	0.957
				Persistently low trajectory vs persistently normal	10.89 (1.91-59.52)	0.009
			Cumulative low-level exposure	Per additional wave of low-level exposure	1.60 (0.94-2.66)	0.079

Emotional-behavioral or neurodevelopmental problem group	Specific emotional-behavioral problem or neurodevelopmental delay	Parent–child interaction measure	Exposure form	Exposure contrast	aOR (95% CI)	*p* value
	Peer problems in the abnormal range	Overall parent–child interaction frequency	Trajectory pattern	Deteriorated trajectory vs persistently normal	1.17 (0.27-3.96)	0.815
				Fluctuating trajectory vs persistently normal	1.64 (0.35-6.23)	0.506
				Improved trajectory vs persistently normal	2.44 (0.65-7.87)	0.175
				Persistently low trajectory vs persistently normal	2.08 (0.55-7.49)	0.274
			Cumulative low-level exposure	Per additional wave of low-level exposure	1.37 (0.89-2.10)	0.154
		Knowledge-learning interaction frequency	Trajectory pattern	Deteriorated trajectory vs persistently normal	1.35 (0.37-4.05)	0.626
				Fluctuating trajectory vs persistently normal	0.28 (0.03-1.32)	0.118
				Improved trajectory vs persistently normal	0.82 (0.24-2.33)	0.721
				Persistently low trajectory vs persistently normal	2.20 (0.53-8.25)	0.267
			Cumulative low-level exposure	Per additional wave of low-level exposure	1.08 (0.67-1.71)	0.735
		Story-reading interaction frequency	Trajectory pattern	Deteriorated trajectory vs persistently normal	0.92 (0.17-3.44)	0.909
				Fluctuating trajectory vs persistently normal	1.67 (0.45-5.12)	0.414
				Improved trajectory vs persistently normal	0.80 (0.23-2.30)	0.696
				Persistently low trajectory vs persistently normal	0.41 (0.00-3.82)	0.507

**Emotional-behavioral or neurodevelopmental problem group**	**Specific emotional-behavioral problem or neurodevelopmental delay**	**Parent–child interaction measure**	**Exposure form**	**Exposure contrast**	**aOR (95% CI)**	***p* value**
			Cumulative low-level exposure	Per additional wave of low-level exposure	0.84 (0.46-1.42)	0.534
		Physical/cultural interaction frequency	Trajectory pattern	Deteriorated trajectory vs persistently normal	0.48 (0.12-1.49)	0.217
				Fluctuating trajectory vs persistently normal	1.45 (0.48-4.04)	0.497
				Improved trajectory vs persistently normal	0.64 (0.07-2.87)	0.600
				Persistently low trajectory vs persistently normal	1.41 (0.24-5.98)	0.674
			Cumulative low-level exposure	Per additional wave of low-level exposure	0.95 (0.58-1.51)	0.843
		Environmental interaction frequency	Trajectory pattern	Deteriorated trajectory vs persistently normal	1.24 (0.22-4.96)	0.782
				Fluctuating trajectory vs persistently normal	1.99 (0.53-6.41)	0.287
				Improved trajectory vs persistently normal	2.00 (0.62-5.63)	0.228
				Persistently low trajectory vs persistently normal	5.97 (0.89-33.02)	0.063
			Cumulative low-level exposure	Per additional wave of low-level exposure	1.43 (0.87-2.31)	0.155
	Prosocial behavior difficulties in the abnormal range	Overall parent–child interaction frequency	Trajectory pattern	Deteriorated trajectory vs persistently normal	1.94 (0.53-6.13)	0.298
				Fluctuating trajectory vs persistently normal	2.18 (0.48-8.06)	0.292
				Improved trajectory vs persistently normal	1.56 (0.28-5.93)	0.568

Emotional-behavioral or neurodevelopmental problem group	Specific emotional-behavioral problem or neurodevelopmental delay	Parent–child interaction measure	Exposure form	Exposure contrast	aOR (95% CI)	*p* value
				Persistently low trajectory vs persistently normal	3.43 (0.96-11.98)	0.058
			Cumulative low-level exposure	Per additional wave of low-level exposure	1.49 (0.97-2.27)	0.069
		Knowledge-learning interaction frequency	Trajectory pattern	Deteriorated trajectory vs persistently normal	0.82 (0.15-2.95)	0.788
				Fluctuating trajectory vs persistently normal	0.36 (0.04-1.64)	0.208
				Improved trajectory vs persistently normal	1.15 (0.37-3.07)	0.799
				Persistently low trajectory vs persistently normal	3.22 (0.85-11.52)	0.083
			Cumulative low-level exposure	Per additional wave of low-level exposure	1.50 (0.96-2.33)	0.075
		Story-reading interaction frequency	Trajectory pattern	Deteriorated trajectory vs persistently normal	1.13 (0.21-4.17)	0.873
				Fluctuating trajectory vs persistently normal	1.01 (0.19-3.65)	0.984
				Improved trajectory vs persistently normal	1.68 (0.61-4.28)	0.306
				Persistently low trajectory vs persistently normal	0.52 (0.00-4.74)	0.637
			Cumulative low-level exposure	Per additional wave of low-level exposure	1.18 (0.70-1.89)	0.525
		Physical/cultural interaction frequency	Trajectory pattern	Deteriorated trajectory vs persistently normal	0.96 (0.33-2.49)	0.930
				Fluctuating trajectory vs persistently normal	0.66 (0.15-2.23)	0.525

**Emotional-behavioral or neurodevelopmental problem group**	**Specific emotional-behavioral problem or neurodevelopmental delay**	**Parent–child interaction measure**	**Exposure form**	**Exposure contrast**	**aOR (95% CI)**	***p* value**
				Improved trajectory vs persistently normal	0.61 (0.06-2.75)	0.564
				Persistently low trajectory vs persistently normal	1.43 (0.25-5.99)	0.655
			Cumulative low-level exposure	Per additional wave of low-level exposure	0.98 (0.59-1.56)	0.939
		Environmental interaction frequency	Trajectory pattern	Deteriorated trajectory vs persistently normal	1.24 (0.29-4.18)	0.749
				Fluctuating trajectory vs persistently normal	0.62 (0.11-2.24)	0.490
				Improved trajectory vs persistently normal	0.82 (0.20-2.49)	0.744
				Persistently low trajectory vs persistently normal	0.48 (0.00-4.62)	0.591
			Cumulative low-level exposure	Per additional wave of low-level exposure	0.85 (0.46-1.45)	0.576
Neurodevelopmental delay	Communication delay	Overall parent–child interaction frequency	Trajectory pattern	Deteriorated trajectory vs persistently normal	0.28 (0.00-2.79)	0.326
				Fluctuating trajectory vs persistently normal	1.32 (0.11-9.09)	0.803
				Improved trajectory vs persistently normal	1.58 (0.14-9.72)	0.663
				Persistently low trajectory vs persistently normal	1.57 (0.22-10.95)	0.649
			Cumulative low-level exposure	Per additional wave of low-level exposure	1.14 (0.58-2.23)	0.708
		Knowledge-learning interaction frequency	Trajectory pattern	Deteriorated trajectory vs persistently normal	1.29 (0.12-7.63)	0.807

**Emotional-behavioral or neurodevelopmental problem group**	**Specific emotional-behavioral problem or neurodevelopmental delay**	**Parent–child interaction measure**	**Exposure form**	**Exposure contrast**	**aOR (95% CI)**	***p* value**
				Fluctuating trajectory vs persistently normal	2.00 (0.36-9.58)	0.408
				Improved trajectory vs persistently normal	1.18 (0.20-5.22)	0.836
				Persistently low trajectory vs persistently normal	1.34 (0.12-9.22)	0.785
			Cumulative low-level exposure	Per additional wave of low-level exposure	0.96 (0.47-1.84)	0.909
		Story-reading interaction frequency	Trajectory pattern	Deteriorated trajectory vs persistently normal	1.05 (0.10-6.24)	0.959
				Fluctuating trajectory vs persistently normal	1.11 (0.11-5.97)	0.914
				Improved trajectory vs persistently normal	1.25 (0.25-5.19)	0.767
				Persistently low trajectory vs persistently normal	1.04 (0.01-11.00)	0.981
			Cumulative low-level exposure	Per additional wave of low-level exposure	0.93 (0.39-1.90)	0.856
		Physical/cultural interaction frequency	Trajectory pattern	Deteriorated trajectory vs persistently normal	0.85 (0.13-3.94)	0.839
				Fluctuating trajectory vs persistently normal	0.49 (0.04-3.09)	0.474
				Improved trajectory vs persistently normal	1.61 (0.15-9.59)	0.650
				Persistently low trajectory vs persistently normal	2.37 (0.20-16.85)	0.441
			Cumulative low-level exposure	Per additional wave of low-level exposure	0.96 (0.42-1.94)	0.918

Emotional-behavioral or neurodevelopmental problem group	Specific emotional-behavioral problem or neurodevelopmental delay	Parent–child interaction measure	Exposure form	Exposure contrast	aOR (95% CI)	*p* value
		Environmental interaction frequency	Trajectory pattern	Deteriorated trajectory vs persistently normal	0.92 (0.08-5.41)	0.936
				Fluctuating trajectory vs persistently normal	0.86 (0.14-3.82)	0.847
				Improved trajectory vs persistently normal	0.78 (0.08-4.01)	0.793
				Persistently low trajectory vs persistently normal	1.24 (0.01-15.79)	0.895
			Cumulative low-level exposure	Per additional wave of low-level exposure	0.69 (0.25-1.55)	0.385
	Gross motor delay	Overall parent–child interaction frequency	Trajectory pattern	Deteriorated trajectory vs persistently normal	1.50 (0.24-7.52)	0.640
				Fluctuating trajectory vs persistently normal	0.38 (0.00-4.85)	0.510
				Improved trajectory vs persistently normal	2.80 (0.44-13.88)	0.249
				Persistently low trajectory vs persistently normal	0.65 (0.05-5.65)	0.709
			Cumulative low-level exposure	Per additional wave of low-level exposure	1.23 (0.61-2.42)	0.553
		Knowledge-learning interaction frequency	Trajectory pattern	Deteriorated trajectory vs persistently normal	1.26 (0.12-6.77)	0.817
				Fluctuating trajectory vs persistently normal	0.83 (0.07-5.17)	0.858
				Improved trajectory vs persistently normal	0.70 (0.07-3.65)	0.701
				Persistently low trajectory vs persistently normal	1.71 (0.15-12.11)	0.620

Emotional-behavioral or neurodevelopmental problem group	Specific emotional-behavioral problem or neurodevelopmental delay	Parent–child interaction measure	Exposure form	Exposure contrast	aOR (95% CI)	*p* value
			Cumulative low-level exposure	Per additional wave of low-level exposure	0.97 (0.42-1.99)	0.928
		Story-reading interaction frequency	Trajectory pattern	Deteriorated trajectory vs persistently normal	1.96 (0.17-12.41)	0.534
				Fluctuating trajectory vs persistently normal	4.25 (0.85-18.35)	0.075
				Improved trajectory vs persistently normal	0.32 (0.00-3.10)	0.384
				Persistently low trajectory vs persistently normal	1.68 (0.01-18.86)	0.753
			Cumulative low-level exposure	Per additional wave of low-level exposure	1.22 (0.50-2.52)	0.633
		Physical/cultural interaction frequency	Trajectory pattern	Deteriorated trajectory vs persistently normal	1.63 (0.33-6.98)	0.528
				Fluctuating trajectory vs persistently normal	0.96 (0.08-6.40)	0.966
				Improved trajectory vs persistently normal	0.66 (0.00-6.98)	0.774
				Persistently low trajectory vs persistently normal	2.70 (0.22-20.62)	0.392
			Cumulative low-level exposure	Per additional wave of low-level exposure	1.13 (0.49-2.35)	0.763
		Environmental interaction frequency	Trajectory pattern	Deteriorated trajectory vs persistently normal	0.38 (0.00-4.14)	0.489
				Fluctuating trajectory vs persistently normal	2.08 (0.33-10.64)	0.409
				Improved trajectory vs persistently normal	1.06 (0.10-5.51)	0.952

Emotional-behavioral or neurodevelopmental problem group	Specific emotional-behavioral problem or neurodevelopmental delay	Parent–child interaction measure	Exposure form	Exposure contrast	aOR (95% CI)	*p* value
				Persistently low trajectory vs persistently normal	0.98 (0.01-13.56)	0.989
			Cumulative low-level exposure	Per additional wave of low-level exposure	0.68 (0.21-1.60)	0.411
	Fine motor delay	Overall parent–child interaction frequency	Trajectory pattern	Deteriorated trajectory vs persistently normal	1.04 (0.23-3.74)	0.958
				Fluctuating trajectory vs persistently normal	0.93 (0.15-4.23)	0.927
				Improved trajectory vs persistently normal	1.04 (0.17-4.36)	0.965
				Persistently low trajectory vs persistently normal	0.39 (0.06-2.10)	0.286
			Cumulative low-level exposure	Per additional wave of low-level exposure	0.88 (0.51-1.48)	0.634
		Knowledge-learning interaction frequency	Trajectory pattern	Deteriorated trajectory vs persistently normal	1.07 (0.19-4.19)	0.934
				Fluctuating trajectory vs persistently normal	1.77 (0.48-5.79)	0.374
				Improved trajectory vs persistently normal	0.64 (0.12-2.32)	0.520
				Persistently low trajectory vs persistently normal	0.26 (0.00-2.47)	0.289
			Cumulative low-level exposure	Per additional wave of low-level exposure	0.64 (0.33-1.15)	0.145
		Story-reading interaction frequency	Trajectory pattern	Deteriorated trajectory vs persistently normal	1.13 (0.19-4.61)	0.882
				Fluctuating trajectory vs persistently normal	1.63 (0.36-5.69)	0.492

Emotional-behavioral or neurodevelopmental problem group	Specific emotional-behavioral problem or neurodevelopmental delay	Parent–child interaction measure	Exposure form	Exposure contrast	aOR (95% CI)	*p* value
				Improved trajectory vs persistently normal	0.30 (0.03-1.39)	0.135
				Persistently low trajectory vs persistently normal	0.46 (0.00-4.55)	0.574
			Cumulative low-level exposure	Per additional wave of low-level exposure	0.82 (0.39-1.51)	0.544
		Physical/cultural interaction frequency	Trajectory pattern	Deteriorated trajectory vs persistently normal	1.56 (0.50-4.49)	0.429
				Fluctuating trajectory vs persistently normal	0.62 (0.10-2.63)	0.539
				Improved trajectory vs persistently normal	0.90 (0.09-4.45)	0.908
				Persistently low trajectory vs persistently normal	0.44 (0.00-4.35)	0.550
			Cumulative low-level exposure	Per additional wave of low-level exposure	0.77 (0.39-1.39)	0.396
		Environmental interaction frequency	Trajectory pattern	Deteriorated trajectory vs persistently normal	0.70 (0.07-3.62)	0.701
				Fluctuating trajectory vs persistently normal	1.62 (0.43-5.36)	0.455
				Improved trajectory vs persistently normal	0.96 (0.17-3.53)	0.953
				Persistently low trajectory vs persistently normal	0.73 (0.01-8.09)	0.836
			Cumulative low-level exposure	Per additional wave of low-level exposure	0.68 (0.30-1.31)	0.264
	Problem-solving delay	Overall parent–child interaction frequency	Trajectory pattern	Deteriorated trajectory vs persistently normal	0.17 (0.00-1.55)	0.135

Emotional-behavioral or neurodevelopmental problem group	Specific emotional-behavioral problem or neurodevelopmental delay	Parent–child interaction measure	Exposure form	Exposure contrast	aOR (95% CI)	*p* value
				Fluctuating trajectory vs persistently normal	0.61 (0.06-3.54)	0.611
				Improved trajectory vs persistently normal	1.39 (0.24-5.81)	0.684
				Persistently low trajectory vs persistently normal	1.15 (0.23-5.47)	0.860
			Cumulative low-level exposure	Per additional wave of low-level exposure	0.99 (0.55-1.71)	0.970
		Knowledge-learning interaction frequency	Trajectory pattern	Deteriorated trajectory vs persistently normal	0.56 (0.05-2.83)	0.518
				Fluctuating trajectory vs persistently normal	0.75 (0.13-3.11)	0.715
				Improved trajectory vs persistently normal	0.95 (0.22-3.22)	0.936
				Persistently low trajectory vs persistently normal	0.69 (0.06-4.06)	0.704
			Cumulative low-level exposure	Per additional wave of low-level exposure	0.87 (0.45-1.55)	0.639
		Story-reading interaction frequency	Trajectory pattern	Deteriorated trajectory vs persistently normal	1.70 (0.29-7.08)	0.516
				Fluctuating trajectory vs persistently normal	0.94 (0.10-4.53)	0.945
				Improved trajectory vs persistently normal	1.12 (0.26-3.89)	0.871
				Persistently low trajectory vs persistently normal	0.64 (0.00-6.47)	0.762
			Cumulative low-level exposure	Per additional wave of low-level exposure	0.80 (0.36-1.54)	0.537

**Emotional-behavioral or neurodevelopmental problem group**	**Specific emotional-behavioral problem or neurodevelopmental delay**	**Parent–child interaction measure**	**Exposure form**	**Exposure contrast**	**aOR (95% CI)**	***p* value**
		Physical/cultural interaction frequency	Trajectory pattern	Deteriorated trajectory vs persistently normal	0.87 (0.20-2.99)	0.837
				Fluctuating trajectory vs persistently normal	0.49 (0.05-2.54)	0.423
				Improved trajectory vs persistently normal	1.11 (0.11-5.43)	0.910
				Persistently low trajectory vs persistently normal	2.66 (0.42-12.85)	0.269
			Cumulative low-level exposure	Per additional wave of low-level exposure	1.03 (0.55-1.82)	0.916
		Environmental interaction frequency	Trajectory pattern	Deteriorated trajectory vs persistently normal	1.06 (0.10-5.43)	0.950
				Fluctuating trajectory vs persistently normal	1.53 (0.34-5.66)	0.553
				Improved trajectory vs persistently normal	0.66 (0.07-3.06)	0.635
				Persistently low trajectory vs persistently normal	3.68 (0.32-24.52)	0.256
			Cumulative low-level exposure	Per additional wave of low-level exposure	1.05 (0.51-1.96)	0.889
	Personal-social delay	Overall parent–child interaction frequency	Trajectory pattern	Deteriorated trajectory vs persistently normal	0.71 (0.07-3.96)	0.721
				Fluctuating trajectory vs persistently normal	0.33 (0.00-3.61)	0.418
				Improved trajectory vs persistently normal	2.18 (0.36-9.84)	0.361
				Persistently low trajectory vs persistently normal	0.51 (0.04-3.96)	0.532

**Emotional-behavioral or neurodevelopmental problem group**	**Specific emotional-behavioral problem or neurodevelopmental delay**	**Parent–child interaction measure**	**Exposure form**	**Exposure contrast**	**aOR (95% CI)**	***p* value**
			Cumulative low-level exposure	Per additional wave of low-level exposure	0.78 (0.36-1.55)	0.487
		Knowledge-learning interaction frequency	Trajectory pattern	Deteriorated trajectory vs persistently normal	1.13 (0.11-5.78)	0.898
				Fluctuating trajectory vs persistently normal	1.36 (0.22-6.17)	0.715
				Improved trajectory vs persistently normal	0.67 (0.07-3.17)	0.644
				Persistently low trajectory vs persistently normal	0.47 (0.00-5.06)	0.592
			Cumulative low-level exposure	Per additional wave of low-level exposure	0.69 (0.27-1.46)	0.350
		Story-reading interaction frequency	Trajectory pattern	Deteriorated trajectory vs persistently normal	3.69 (0.59-17.62)	0.148
				Fluctuating trajectory vs persistently normal	1.97 (0.19-10.43)	0.501
				Improved trajectory vs persistently normal	1.70 (0.28-7.56)	0.525
				Persistently low trajectory vs persistently normal	1.87 (0.01-20.12)	0.705
			Cumulative low-level exposure	Per additional wave of low-level exposure	1.34 (0.60-2.66)	0.446
		Physical/cultural interaction frequency	Trajectory pattern	Deteriorated trajectory vs persistently normal	2.30 (0.49-9.23)	0.275
				Fluctuating trajectory vs persistently normal	1.41 (0.13-9.02)	0.742
				Improved trajectory vs persistently normal	0.85 (0.01-8.55)	0.914

Emotional-behavioral or neurodevelopmental problem group	Specific emotional-behavioral problem or neurodevelopmental delay	Parent–child interaction measure	Exposure form	Exposure contrast	aOR (95% CI)	*p* value
				Persistently low trajectory vs persistently normal	7.98 (1.07-51.32)	0.043
			Cumulative low-level exposure	Per additional wave of low-level exposure	1.50 (0.73-2.89)	0.260
		Environmental interaction frequency	Trajectory pattern	Deteriorated trajectory vs persistently normal	0.36 (0.00-3.49)	0.446
				Fluctuating trajectory vs persistently normal	0.25 (0.00-2.33)	0.266
				Improved trajectory vs persistently normal	0.82 (0.08-3.89)	0.823
				Persistently low trajectory vs persistently normal	3.92 (0.33-28.25)	0.242
			Cumulative low-level exposure	Per additional wave of low-level exposure	0.85 (0.30-1.88)	0.705

Note. Values are adjusted odds ratios from Firth penalized logistic regression models.

Supplementary Table S6. Complete adjusted estimates for post hoc analyses of single-wave low-level overall parent–child interaction frequency across emotional-behavioral and neurodevelopmental outcomes.

Outcome	Exposure wave	aOR (95% CI)	*p* value
Abnormal total difficulties	Wave 1	1.35 (0.53-3.45)	0.524
	Wave 2	**3.31 (1.31-8.38)**	**0.011**
	Wave 3	1.91 (0.76-4.78)	0.166
Emotional symptoms in the abnormal range	Wave 1	0.76 (0.26-2.21)	0.612
	Wave 2	1.46 (0.53-4.03)	0.466
	Wave 3	0.76 (0.27-2.17)	0.614
Conduct problems in the abnormal range	Wave 1	1.48 (0.59-3.72)	0.405
	Wave 2	**2.86 (1.15-7.14)**	**0.024**
	Wave 3	2.08 (0.83-5.24)	0.120
Hyperactivity/inattention in the abnormal range	Wave 1	1.39 (0.48-4.04)	0.541
	Wave 2	**4.18 (1.45-12.09)**	**0.008**
	Wave 3	1.27 (0.44-3.67)	0.656
Peer problems in the abnormal range	Wave 1	2.04 (0.77-5.36)	0.150
	Wave 2	2.08 (0.79-5.50)	0.139
	Wave 3	1.15 (0.43-3.09)	0.781
Prosocial behavior in the abnormal range	Wave 1	1.90 (0.72-5.01)	0.192
	Wave 2	1.81 (0.68-4.80)	0.233
	Wave 3	2.24 (0.85-5.88)	0.103
Screening-positive delay in any neurodevelopmental domain	Wave 1	0.95 (0.35-2.56)	0.919
	Wave 2	1.16 (0.44-3.12)	0.761
	Wave 3	0.51 (0.18-1.42)	0.197
Communication delay	Wave 1	2.74 (0.57-13.26)	0.210
	Wave 2	0.83 (0.17-4.01)	0.818
	Wave 3	0.89 (0.19-4.24)	0.881
Gross motor delay	Wave 1	0.96 (0.18-5.05)	0.962
	Wave 2	4.33 (0.91-20.61)	0.065
	Wave 3	0.63 (0.12-3.36)	0.584
Fine motor delay	Wave 1	0.51 (0.14-1.86)	0.309
	Wave 2	1.55 (0.48-5.03)	0.468
	Wave 3	0.59 (0.17-2.03)	0.403
Problem-solving delay	Wave 1	1.31 (0.37-4.60)	0.678
	Wave 2	1.61 (0.47-5.58)	0.450
	Wave 3	0.39 (0.10-1.56)	0.183
Personal-social delay	Wave 1	1.06 (0.21-5.28)	0.944
	Wave 2	0.58 (0.10-3.38)	0.544
	Wave 3	0.34 (0.06-2.06)	0.240

Note. Values are adjusted odds ratios from post hoc single-wave logistic regression models using the same 419 children and primary covariates as the main analysis. For each wave, low-level exposure was compared with non-low-level exposure, and each model included one wave-specific exposure indicator. These models compare wave-specific measurements and should not be interpreted as causal effects attributable to a particular wave. p values are nominal and were not adjusted for multiple comparisons. Bold values indicate $p < 0.05$.

Supplementary Table S7. Complete adjusted estimates for post hoc analyses of single-wave low-level content-domain and activity-level exposures in relation to SDQ and ASQ-3 outcomes.

Outcome group	Parent–child interaction measure	Outcome	Exposure wave	aOR (95% CI)	*p* value
Emotional and behavioral problems	Story-reading domain	Abnormal total difficulties	Wave 1	0.89 (0.35-2.27)	0.803
			Wave 2	**3.51 (1.45-8.51)**	**0.005**
			Wave 3	1.50 (0.54-4.15)	0.436
		Conduct problems in the abnormal range	Wave 1	1.36 (0.57-3.22)	0.487
			Wave 2	2.25 (0.89-5.66)	0.086
			Wave 3	1.29 (0.46-3.63)	0.625
		Hyperactivity/inattention in the abnormal range	Wave 1	1.38 (0.51-3.76)	0.526
			Wave 2	**3.51 (1.30-9.49)**	**0.013**
			Wave 3	1.13 (0.34-3.78)	0.845
	Environmental interaction domain	Abnormal total difficulties	Wave 1	1.53 (0.62-3.78)	0.356
			Wave 2	2.00 (0.79-5.11)	0.146
			Wave 3	1.88 (0.69-5.13)	0.215
		Conduct problems in the abnormal range	Wave 1	1.98 (0.84-4.68)	0.119
			Wave 2	1.56 (0.61-3.96)	0.353
			Wave 3	1.40 (0.51-3.85)	0.511
		Hyperactivity/inattention in the abnormal range	Wave 1	1.46 (0.51-4.16)	0.476
			Wave 2	1.83 (0.63-5.30)	0.267
			Wave 3	2.72 (0.92-8.06)	0.070
	Physical/cultural domain	Hyperactivity/inattention in the abnormal range	Wave 1	2.39 (0.87-6.57)	0.092
			Wave 2	0.55 (0.17-1.75)	0.313
			Wave 3	**3.23 (1.32-7.88)**	**0.010**
	Reading picture books	Abnormal total difficulties	Wave 1	1.11 (0.34-3.63)	0.869
			Wave 2	**14.17 (3.77-53.26)**	**<0.001**
			Wave 3	0.67 (0.08-5.60)	0.713
		Hyperactivity/inattention in the abnormal range	Wave 1	1.01 (0.26-3.98)	0.986
			Wave 2	**8.55 (1.99-36.77)**	**0.004**
			Wave 3	2.50 (0.46-13.46)	0.285
	Learning about nature/animals/plants	Abnormal total difficulties	Wave 1	1.43 (0.45-4.51)	0.539
			Wave 2	**3.97 (1.48-10.67)**	**0.006**
			Wave 3	1.47 (0.45-4.80)	0.523
		Conduct problems in the abnormal range	Wave 1	1.78 (0.61-5.24)	0.294
			Wave 2	2.49 (0.88-7.04)	0.085
			Wave 3	2.46 (0.84-7.19)	0.099
		Hyperactivity/inattention in the abnormal range	Wave 1	2.07 (0.63-6.80)	0.229
			Wave 2	**3.95 (1.28-12.25)**	**0.017**
			Wave 3	2.81 (0.88-8.92)	0.081
		Peer problems in the abnormal range	Wave 1	1.56 (0.48-5.01)	0.457
			Wave 2	**3.80 (1.32-10.92)**	**0.013**
			Wave 3	1.66 (0.50-5.57)	0.411
Neurodevelopmental delay	Physical/cultural domain	Personal-social delay	Wave 1	1.30 (0.25-6.88)	0.759
			Wave 2	1.30 (0.28-5.97)	0.735
			Wave 3	2.55 (0.70-9.35)	0.157
	Physical activity	Screening-positive delay in any neurodevelopmental domain	Wave 1	1.93 (0.76-4.91)	0.169
			Wave 2	0.91 (0.35-2.35)	0.848
			Wave 3	**2.94 (1.31-6.58)**	**0.009**
		Communication delay	Wave 1	**4.29 (1.08-17.03)**	**0.039**
			Wave 2	2.91 (0.86-9.85)	0.086
			Wave 3	2.78 (0.75-10.22)	0.125

Note. *Analyses were limited to exposure-outcome pairs with nominal $p < 0.05$ in the corresponding longitudinal analyses and assessed whether the associations were also observed at a single wave. Models used the primary covariate set. For each wave, low-level exposure was compared with non-low-level exposure. p values are nominal and were not adjusted for multiple comparisons. Large estimates based on very small low-exposure groups should be interpreted cautiously. Bold values indicate $p < 0.05$.*
